# mTORC1‐USP30‐LEF1 Cascade Regulates Cancer Stemness and Malignant Progression Through Mitonuclear Crosstalk

**DOI:** 10.1002/mco2.70499

**Published:** 2025-11-24

**Authors:** Xiaolin Li, Haowei Zhang, Jia Li, Cheng Luo, Zijian Yang, Jin Cai, Li Xia, Yapei Jiang, Ruonan Wang, Hongli Zeng, Yuetong Li, Haitao Yang, Tong Gao, Weidong Xie, Yaou Zhang, Naihan Xu

**Affiliations:** ^1^ School of Food and Drug Shenzhen Polytechnic University Shenzhen China; ^2^ State Key Laboratory of Chemical Oncogenomics Institute of Biopharmaceutical and Health Engineering Tsinghua Shenzhen International Graduate School Tsinghua University Shenzhen China; ^3^ Department of Breast and Thyroid Surgery Peking University Shenzhen Hospital Shenzhen China

**Keywords:** chemoresistance, deubiquitination, stemness, TCF/LEF1, USP30, WNT

## Abstract

USP30, a ubiquitin‐specific protease, primarily characterized as a mitochondrial deubiquitinase regulating mitophagy, has not been previously reported to have nuclear functions. In this study, we demonstrate that USP30 is present in both mitochondrial and nuclear compartments. Nutrient deprivation triggers USP30 nuclear translocation via an N‐terminal nuclear localization signal (NLS), mediated through suppression of mTORC1‐dependent phosphorylation at serine 104, a modification constraining nuclear entry. Nuclear USP30 acts as a tumor suppressor by inhibiting cancer stemness and chemoresistance in triple‐negative breast cancer (TNBC) cells. Mechanistically, USP30 directly interacts with and deubiquitinates the transcription factor TCF/LEF1 at K379 and K382 residues, disrupting recruitment of CBP/P300 co‐activators to the β‐catenin/LEF1 complex. This abolishes β‐catenin/LEF1 transactivation and suppresses WNT signaling. Clinically, USP30 is downregulated in TNBC and cancer stem cells (CSCs), with notably reduced nuclear levels in cancer tissues. Overexpression of nuclear USP30 markedly reduces lung metastatic burden in TNBC mouse models. These findings uncover a novel role for nuclear USP30 in regulating cancer stemness and suggest that targeting the dynamic relocalization of USP30 from mitochondria to the nucleus could offer new therapeutic strategies for breast cancer metastasis.

## Graphical abstract

Nutrient deprivation triggers USP30 nuclear translocation via an N‐terminal nuclear localization signal (NLS), mediated through suppression of mTORC1‐dependent phosphorylation at serine 104, a modification constraining nuclear entry. Nuclear USP30 deubiquitinates TCF/LEF1 at K379 and K382 residues, disrupting recruitment of CBP/P300 co‐activators to the β‐catenin/LEF1 complex and inhibiting WNT signaling. Nuclear‐localized USP30 suppresses cancer stemness, chemoresistance, and metastasis in TNBC models.

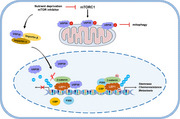

## 1 Introduction

Ubiquitination is a dynamic post‐translational modification (PTM) that regulates virtually cellular processes through covalent attachment of ubiquitin chains to target proteins. This reversible process, counterbalanced by deubiquitinating enzymes (DUBs) [1, 2, 3], governs protein stability, localization, and interaction networks critical for cellular homeostasis [4, 5, 6, 7]. Dysregulation of ubiquitin signaling, particularly through aberrant activity of DUBs, disrupts essential pathways including DNA repair, cell cycle control, and stress responses, contributing to tumorigenesis, metastatic progression, and thereby drug resistance [8, 9, 10, 11]. Among DUBs, ubiquitin‐specific proteases (USPs) family constitutes the largest subclass, comprising over 50 members [1]. A hallmark of USPs is a highly conserved catalytic core featuring histidine and cysteine boxes, often flanked by ubiquitin‐binding, ubiquitin‐like, or zinc‐finger domains [6]. Their catalytic capability primarily depends on a nucleophilic cysteine residue within the catalytic site [12]. Mounting evidence highlights the involvement of USPs in tumorigenesis and cancer progression through the regulation of multiple cancer‐related pathways, positioning them as attractive targets for therapeutic drug development [13, 14].

Mitochondrial function is coordinated through bidirectional communication with the nucleus. While anterograde signaling (nucleus‐to‐mitochondria) regulate mitochondrial biogenesis via nuclear‐encoded genes, retrograde signaling (mitochondria‐to‐nucleus) activates transcriptional reprogramming in response to metabolic stress, damage, or nutrient fluctuations [15]. Remarkably, multiple mitochondrial metabolic enzymes, including hexokinase 2 (HK2), pyruvate dehydrogenase (PDH1), pyruvate carboxylase (PCB), aconitase 2 (ACO2), citrate synthase (CS), fumarase (FH), and isocitrate dehydrogenase 3 (IDH3A), translocate to the nucleus under stress conditions to modulate epigenetic landscapes, DNA repair, and stemness pathways [16, 17, 18, 19, 20]. Similarly, genome‐stabilizing proteins, like telomerase reverse transcriptase (TERT) and DNA helicase RecQ helicase‐like 4 (RECQL4), display stress‐induced dual mitochondrial and nuclear localization, protecting mitochondrial DNA and influencing nuclear functions [12, 21, 22]. This dynamic compartmentalization allows cells to adapt to microenvironmental challenges in cancer to promote survival, stemness, and metastasis.

Ubiquitin‐specific peptidase 30 (USP30) is a deubiquitinase predominantly anchored to the mitochondrial outer membrane via its unique transmembrane domain [7]. It features a C‐terminal USP catalytic domain and preferentially cleaves Lys6‐ and Lys11‐linked polyubiquitin chains [23]. Initially recognized as a negative regulator of Parkin/PINK1‐mediated mitophagy, USP30 inhibition is potentially beneficial for Parkinson's disease by promoting mitochondrial clearance and quality control [24]. USP30 also plays essential roles in pexophagy, Bax/Bak‐dependent apoptosis, and cytotoxic T lymphocytes (CTLs) function [25, 26]. Its pathophysiological significance extends to cancer, where dysregulation of USP30 promotes hepatocellular carcinoma (HCC) through the IKKβ‐USP30‐ACLY lipogenesis axis [27] and drives breast cancer progression via Snail stabilization [28]. Despite these advances, two critical gaps persist: (i) whether USP30 exhibits functional nuclear localization, a feature observed in other mitochondrial proteins, remains unexplored, and (ii) its functional significance beyond mitochondrial contexts is unknown, particularly in aggressive cancers like triple‐negative breast cancer (TNBC), which lacks targeted therapies and frequently exhibits therapy‐resistant stem cell populations.

Given the prevalence of dual‐localization proteins at the mitochondria‐nucleus interface and the established roles of USPs in cancer signaling, we hypothesized that USP30 may exert non‐canonical nuclear functions. In this study, we discovered that USP30 displays both mitochondrial and nuclear localization. Nutrient deprivation facilitates its nuclear translocation, mediated through suppression of mTORC1‐mediated phosphorylation at serine 104. Within the nucleus, USP30 exerts tumor‐suppressive effects by inhibiting cancer stemness and chemoresistance in TNBC cells. Mechanistically, USP30 interacts with the transcription factor TCF/LEF1 and deubiquitinates it at residues K379 and K382. This deubiquitination event prevents recruitment of co‐activators CBP/P300 to the β‐catenin/LEF1 complex, thereby inhibiting WNT‐driven stemness, chemoresistance, and metastasis. This work unveils a mitochondria‐nucleus signaling axis centered on USP30 relocalization, providing new mechanistic insights and therapeutic opportunities for TNBC.

## 2 Results

### 2.1 USP30 Exhibits Dual Localization in Both Mitochondria and the Nucleus

**FIGURE 1:**
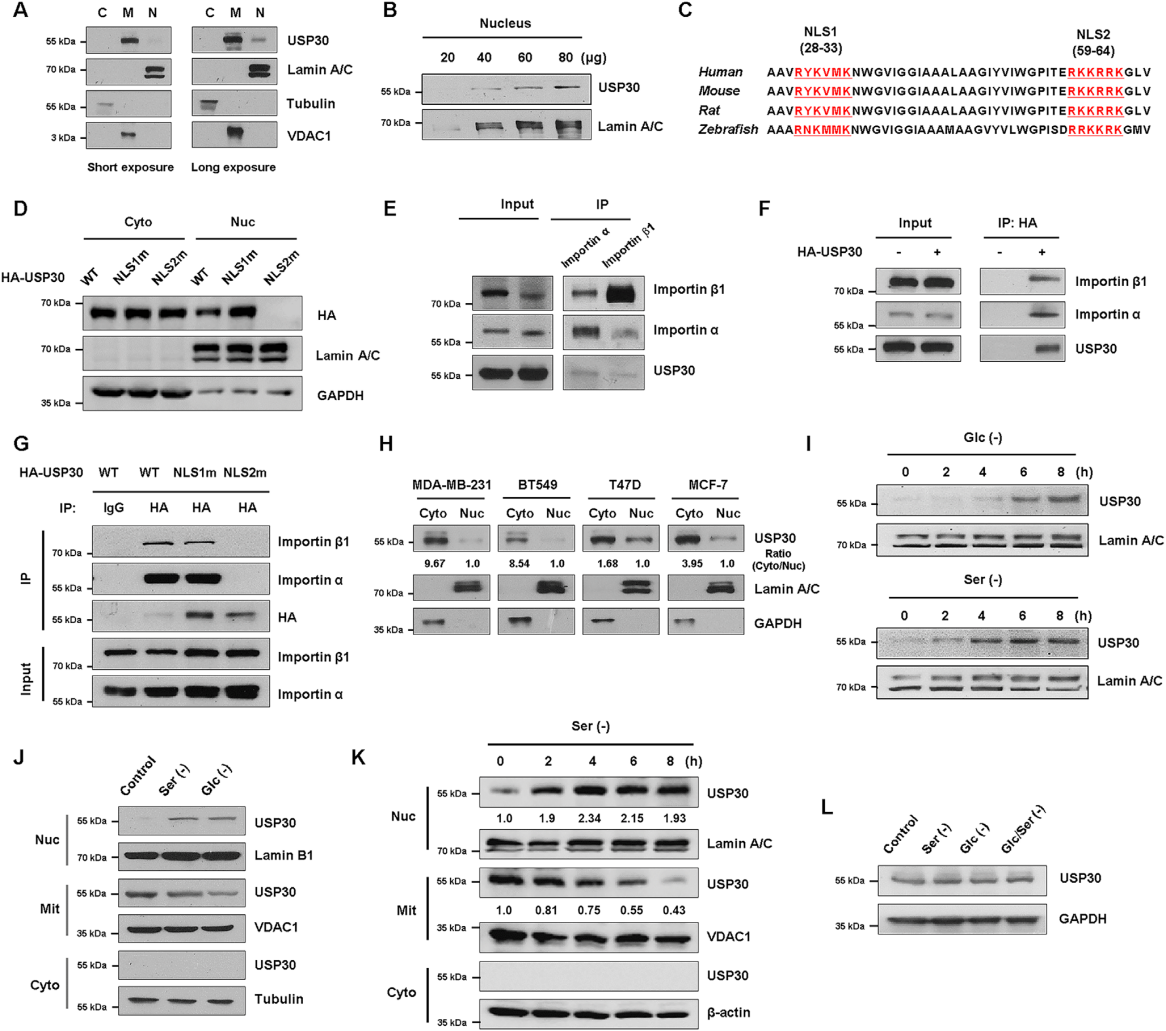
**The nuclear localization of USP30**. (A) Immunoblots of endogenous USP30 in different subcellular fractions of MDA‐MB‐231 cells with varying exposure times. C, cytoplasm; M, mitochondria; N, nucleus. (B) Immunoblots of endogenous USP30 in nuclear fractions of MDA‐MB‐231 cells. (C) NLS1 and NLS2: two conserved putative bipartite nuclear localization signals. (D) Immunoblots of USP30 in cytoplasmic and nuclear fractions of MDA‐MB‐231 cells after transfection with HA‐USP30, HA‐USP30‐NLS1 mutant, or HA‐USP30‐NLS2 mutant plasmids. (E) Co‐IP assay showed the interaction between endogenous importinα, importinβ1, and USP30 in HEK293 cells. (F) Co‐IP assay showed the interaction between exogenous HA‐USP30 and importinα/β1 in HEK293 cells. (G) Co‐IP assay showed that HA‐USP30 and NLS1 mutant, but not the NLS2 mutant, interacted with both importinα and β1 in HEK293 cells. (H) Immunoblots of endogenous USP30 in different subcellular fractions of MDA‐MB‐231, BT549, T47D, and MCF7 cells. Cyto, cytoplasm; Nuc, nucleus. (I–K) Spatial and temporal assessment of USP30 localization in MDA‐MB‐231 cells after glucose restriction and serum deprivation by subcellular fractionation and immunoblotting. (L) Immunoblots of total USP30 protein in MDA‐MB‐231 cells after glucose restriction (4 h) and serum deprivation (4 h).

An increasing number of mitochondrial proteins have been reported to reside in the nucleus to control mitochondria‐to‐nucleus communication [29]. Earlier studies demonstrate that USP30 localizes in the mitochondrial outer membrane and peroxisomes owing to its N‐terminal transmembrane domain [30]. To thoroughly investigate the subcellular localization of USP30, we isolated the cytosolic, nuclear, and mitochondrial fractions from TNBC cell MDA‐MB‐231. Immunoblotting analysis showed that USP30 mainly concentrated within the mitochondrial fraction. However, when the exposure time was prolonged or the amounts of nuclear extracts were increased, USP30 was also detected in the nuclear fraction (Figure 1A,B). Analysis of the USP30 protein sequence revealed two conserved putative bipartite nuclear localization signals at N‐terminal regions (Figure 1C). To determine whether these sequences are functional for nuclear localization, we generated NLS1m and NLS2m mutants where the Lys and Arg residues were replaced with uncharged residues. Both immunofluorescence and cytoplasmic/nuclear fractionation assays showed that NLS1m exhibited the same subcellular distribution as wild‐type (WT) USP30, whereas NLS2m was excluded from the nucleus (Figure 1D).

Nuclear transport of NLS‐containing proteins is usually mediated by nuclear transport receptors importin α and importin β1. Immunoprecipitation assay showed that USP30 could bind to both importin α and importin β1 (Figure 1E,F). HA‐tagged WT and NLS1m, but not NLS2m USP30, interacted with both importin α and β1 (Figure 1G). These data suggest that NLS2 is responsible for USP30's nuclear localization, and the nuclear translocation is dependent on the importin α/β1 complex.

We examined the subcellular distribution of USP30 in multiple breast cancer cell lines. Cytoplasmic and nuclear fractionation assay proved that USP30 exhibited dual localization in both cytoplasmic and nuclear fractions. Notably, TNBC cell lines MDA‐MB‐231 and BT549 expressed lower levels of nuclear USP30 than luminal cell lines T47D and MCF‐7 (Figure 1H). The dual localization of USP30 was also confirmed in additional malignant cell lines, including HepG2 (liver cancer) and HeLa (cervical cancer), as well as in the non‐transformed HEK293 cell line. The cytoplasmic‐to‐nuclear USP30 ratio was significantly higher in cancer cells compared to normal counterparts (Figure S1A). Cellular stress is known to trigger the translocation of proteins into the nucleus, serving as a mechanism for signal transduction and regulation of nuclear functions. To investigate the possibility that cellular stress induces the nuclear import of USP30, MDA‐MB‐231 cells were subjected to serum‐ or glucose‐deprivation for varying durations. Subsequently, nuclear fractions were isolated to detect the expression of USP30. Immunoblotting revealed that nutrient depletion facilitated the nuclear translocation of USP30 in a time‐dependent manner (Figure 1I). Concurrently, the amounts of mitochondria‐bound USP30 decreased under nutrient deprivation (Figure 1J,K, Figure S1B), while the overall USP30 levels remained unchanged (Figure 1L). Immunostaining further confirmed that nutrient deprivation promoted the nuclear translocation of USP30 (Figure S1C).

### 2.2 Nutrient Deprivation‐Induced Nuclear Translocation of USP30 Is Regulated by mTORC1‐Dependent Phosphorylation at Serine 104

**FIGURE 2:**
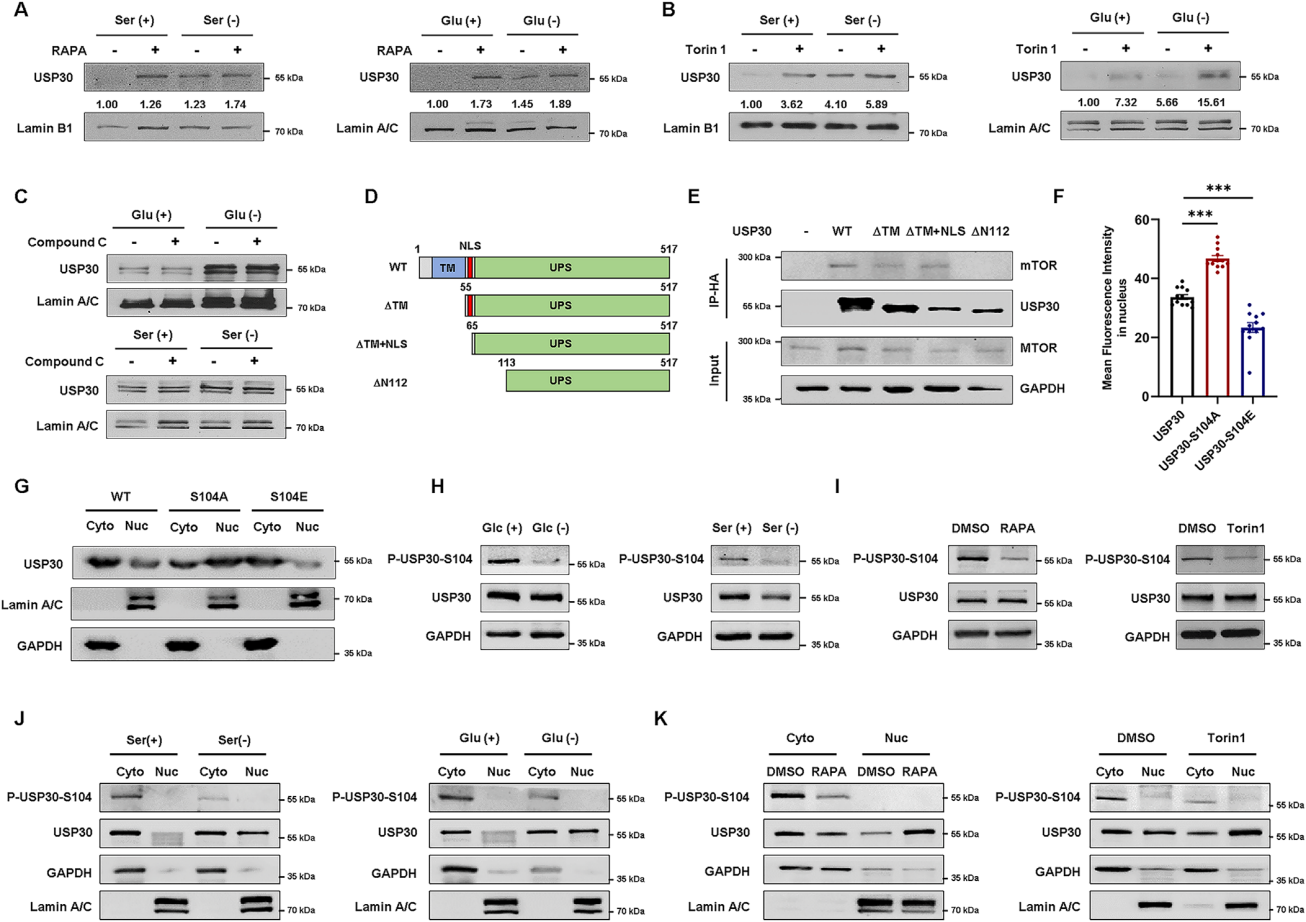
**USP30 nuclear translocation is regulated by mTORC1‐dependent phosphorylation at Serine 104**. (A–C) Nuclear USP30 levels after serum deprivation (4 h) and glucose restriction (4 h) with or without pretreatment with: (A) Rapamycin (50 nM, 12 h), (B) Torin 1 (250 nM, 12 h), or (C) Compound C (5 µM, 12 h). (D) Schematic diagram of USP30 truncation mutations. (E) Co‐IP analysis of the physical interaction between USP30 and mTOR affected by USP30 truncation mutations. (F) Quantification of mean fluorescence intensity of USP30 in the nucleus of MDA‐MB‐231 cells transfected with HA‐USP30, HA‐USP30‐S104A, or HA‐USP30‐S104E plasmids. Data are presented as mean ± SD, and statistical significance was determined by one‐way ANOVA (^∗∗∗^ *p* < 0.001). (G) Western blot analysis of USP30 subcellular localization in HEK293 cells transfected with His‐USP30 or USP30‐S104A/E. (H) Immunoblots of P‐USP30‐S104 in MDA‐MB‐231 cells after glucose restriction (4 h) and serum deprivation (4 h). (I) Immunoblots of P‐USP30‐S104 in MDA‐MB‐231 cells pretreated with or without rapamycin (50 nM, 12 h) or Torin 1 (250 nM, 12 h). (J) The subcellular localization of P‐USP30‐S104S in MDA‐MB‐231 cells after serum deprivation (4 h) and glucose restriction (4 h) deprivation, analyzed by western blotting. (K) The subcellular localization of P‐USP30‐S104S in MDA‐MB‐231 cells pretreated with or without rapamycin (50 nM, 12 h) or Torin 1 (250 nM, 12 h) analyzed by western blotting.

Changes in nutrient levels can be sensed by a highly conserved signaling cascade involving mTOR (mammalian target of rapamycin) kinase, PKA (protein kinase A), and AMPK (AMP‐activated kinase), which collectively adjust cellular physiology [11]. Analysis using USP30 binding protein IP‐MS revealed that USP30 potentially interacted with several protein kinases, such as mTOR, AMPK, PKA, MAPK, CKI, and CKII (Figure S1D). To identify the upstream protein kinases involved in regulating USP30 nuclear translocation, we treated cells with specific inhibitors. Immunoblotting showed that mTOR inhibitors, such as Rapamycin and Torin 1, increased nuclear USP30 levels in cells cultured in complete or glucose/serum‐free medium (Figure 2A,B). In contrast, inhibitors of AMPK, CKI, CKII, PKA, and MAPK did not affect USP30 nuclear translocation (Figure 2C, Figure S1E,F). Co‐immunoprecipitation (Co‐IP) assays demonstrated an interaction between endogenous USP30 and mTOR in MDA‐MB‐231 cells (Figure S1G). Immunoprecipitation experiments with USP30 truncation mutants (ΔTM, ΔTM+NLS, and ΔN112) indicated that the N‐terminal 65–112 amino acids are critical for this interaction (Figure 2D,E). Additionally, we found that the phosphorylation levels of serine/threonine residues in nuclear‐localized USP30 were significantly lower compared to those in cytoplasmic‐localized USP30 (Figure S1H). These results suggest that mTOR kinase may promote USP30's nuclear translocation by regulating its phosphorylation.

To identify the specific mTOR phosphorylation sites on USP30, we analyzed predicted phosphorylation sites in relation to interacting structural domains and mutated the most likely candidate, serine 104. We observed that S104A was more localized in the nucleus than USP30 WT, while S104E exhibited reduced nuclear localization (Figure 2F,G). To further validate the role of mTOR in regulating USP30 S104 phosphorylation, we generated antibodies specific to phosphor‐USP30 Ser104. Immunoblotting results showed that nutrient deprivation and mTOR inhibitors reduced the expression levels of phosphor‐USP30 S104 in MDA‐MB‐231 cells (Figure 2H,I). Analysis of the subcellular distribution of phosphor‐USP30 S104 indicated that it was predominantly cytoplasmic, with reduced expression following nutrient depletion or mTORC1 inhibitor treatment (Figure 2J,K). These results demonstrate that mTORC1‐dependent phosphorylation at serine 104 regulates the nutrient deprivation‐induced nuclear translocation of USP30.

### 2.3 Nuclear USP30 Suppresses Cancer Stemness and Chemoresistance

**FIGURE 3:**
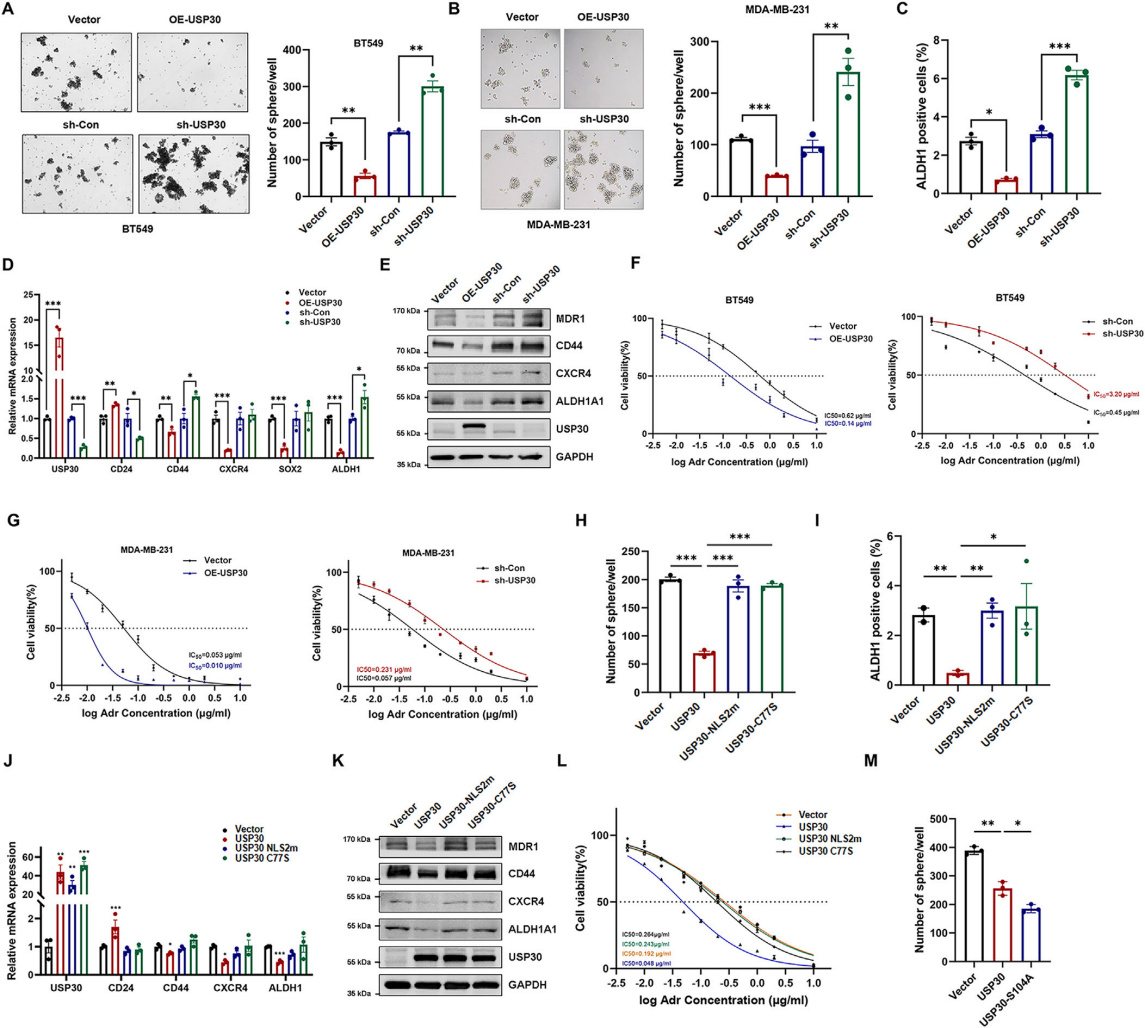
**USP30 Inhibits cancer stemness and chemoresistance in TNBC cells**. (A and B) Sphere formation ability of BT549 and MDA‐MB‐231 cells with USP30 overexpression or knockdown. (C) Assessment of ALDH activity in MDA‐MB‐231 spheres with USP30 overexpression or knockdown by flow cytometry. (D) RT‐qPCR analysis of CSC‐like behavior‐related genes in MDA‐MB‐231 cells with USP30 overexpression or knockdown. (E) Western blot analysis of cancer stemness and chemoresistance markers in MDA‐MB‐231 cells with USP30 overexpression or USP30 knockdown. (F and G) BT549 and MDA‐MB‐231 cells with USP30 overexpression or knockdown were exposed to different concentrations of Adriamycin for 72 h, and the cell viability was determined by CCK‐8. Data were normalized to the vector or sh‐Con control (set as 100%) and are presented as mean ± SD from three independent experiments. (H) Sphere formation in MDA‐MB‐231 cells infected with lentiviruses carrying empty vector, USP30, USP30‐C77S, or USP30‐NLSm. (I) Assessment of ALDH activity in MDA‐MB‐231 spheres infected with lentiviruses carrying empty vector, USP30, USP30‐NLSm, or USP30‐C77S by flow cytometry. (J) RT‐qPCR analysis of CSC‐like behavior‐related genes in MDA‐MB‐231 cells transfected with HA‐USP30, HA‐USP30‐NLS2m, or HA‐USP30‐C77S plasmids. (K) Western blot analysis of cancer stemness and chemoresistance markers in MDA‐MB‐231 cells transfected with HA‐USP30, HA‐USP30‐NLS2m, or HA‐USP30‐C77S plasmids. (L) MDA‐MB‐231 cells infected with lentiviruses carrying empty vector, USP30, USP30‐NLSm, or USP30‐C77S were exposed to different concentrations of Adriamycin for 72 h, and the cell viability was determined by CCK‐8. (M) Comparison of sphere formation in MDA‐MB‐231 cells infected with lentiviruses carrying empty vector, USP30, or USP30‐S104A. Data are presented as mean ± SD from three independent experiments. Statistical significance was determined by one‐way ANOVA (^∗^ *p* < 0.05, ^∗∗^ *p* < 0.01, ^∗∗∗^ *p* < 0.001).

Gene expression correlation analysis revealed that USP30 negatively correlates with the expression of stem cell markers such as MMP2, MMP9, MYC, OCT4(POU5F1), NANOG, ALDH1A3, CD133 (PROM1), EPCAM, CD24, and ITGA6 in breast cancer cohorts (Figure S2A–F). Notably, USP30 was found to be downregulated in TNBC stem‐like cells (Figure S3A). Functional assays in TNBC cell lines BT549 and MDA‐MB‐231 demonstrated that USP30 overexpression suppressed tumor‐sphere formation, whereas its depletion enhanced the CSC‐like properties of these cells (Figure 3A,B and Figure S3B,C). Fluorescence activated cell sorting (FACS) analysis further revealed that USP30 overexpression reduced the proportion of ALDH1‐positive cells in MDA‐MB‐231 stem cell spheres, while USP30 knockdown increased this proportion (Figure 3C and Figure S3F). Reverse transcription‐quantitative PCR (RT‐qPCR) and western blotting results indicated that USP30 overexpression led to decreased expression of stem cell and chemoresistance markers, including CD44, CXCR4, SOX2, ALDH1, and MDR1, while increasing CD24 expression (Figure 3D,E). Additionally, CCK‐8 assays showed that USP30 overexpression increased sensitivity to adriamycin and cisplatin‐induced growth inhibition, in contrast to USP30 knockdown cells, which exhibited reduced drug sensitivity (Figure 3F,G and Figure S3D).

To elucidate the functional significance of USP30 nuclear localization in suppressing cancer stemness and chemoresistance, we performed comprehensive functional analyses comparing WT USP30 with various USP30 mutants. Only WT USP30 significantly inhibited tumor‐sphere formation in MDA‐MB‐231 cells, while both the nuclear‐localization‐deficient NLS2m and catalytic inactive C77S mutants lost this capacity (Figure 3H and Figure S3G). Consistently, flow cytometry analysis revealed that WT USP30, but not the C77S or NLS2m mutants, effectively reduced the ALDH1‐positive cell population (Figure 3I and Figure S3F). RT‐qPCR and western blotting further showed that WT USP30 specifically downregulated the expression of key stem cell markers (CD44, CXCR4, and ALDH1) and chemoresistance‐associated proteins MDR1 (Figure 3J,K). Importantly, only WT USP30 enhanced cellular sensitivity to chemotherapeutic agents adriamycin and cisplatin (Figure 3L and Figure S3E). The enhanced nuclear retention of the S104A mutant correlated with more potent inhibition of tumor stem cell spheroid formation compared to WT USP30 (Figure 3M and Figure S3G). These findings strongly support the nuclear‐localization‐dependent mechanism of USP30's tumor suppressive function.

To systematically investigate the relationship between USP30 subcellular localization and biological function, we generated a series of localization‐specific mutants. Deletion of the transmembrane (TM) domain resulted in cytoplasmic redistribution, while NLS truncation impaired nuclear entry. Conversely, the addition of an exogenous NLS signal enhanced nuclear accumulation (Figure S4A–D). Functional characterization of these mutants revealed that mitochondrial‐localized WT USP30, but not cytoplasmic or nuclear‐localized mutants, effectively inhibited CCCP‐induced mitophagy, as evidenced by preserved mitochondrial membrane proteins (Figure S4E–G). Intriguingly, forced nuclear localization through NLS addition significantly enhanced USP30's ability to suppress cancer stemness and chemoresistance, while cytoplasmic retention or nuclear exclusion abolished these effects (Figure S5A–E). These results indicate that mitochondrial USP30 serves as a negative regulator of mitophagy, whereas nuclear USP30 exerts tumor‐suppressive effects.

### 2.4 TCF/LEF1 Is a Novel Substrate of USP30

**FIGURE 4:**
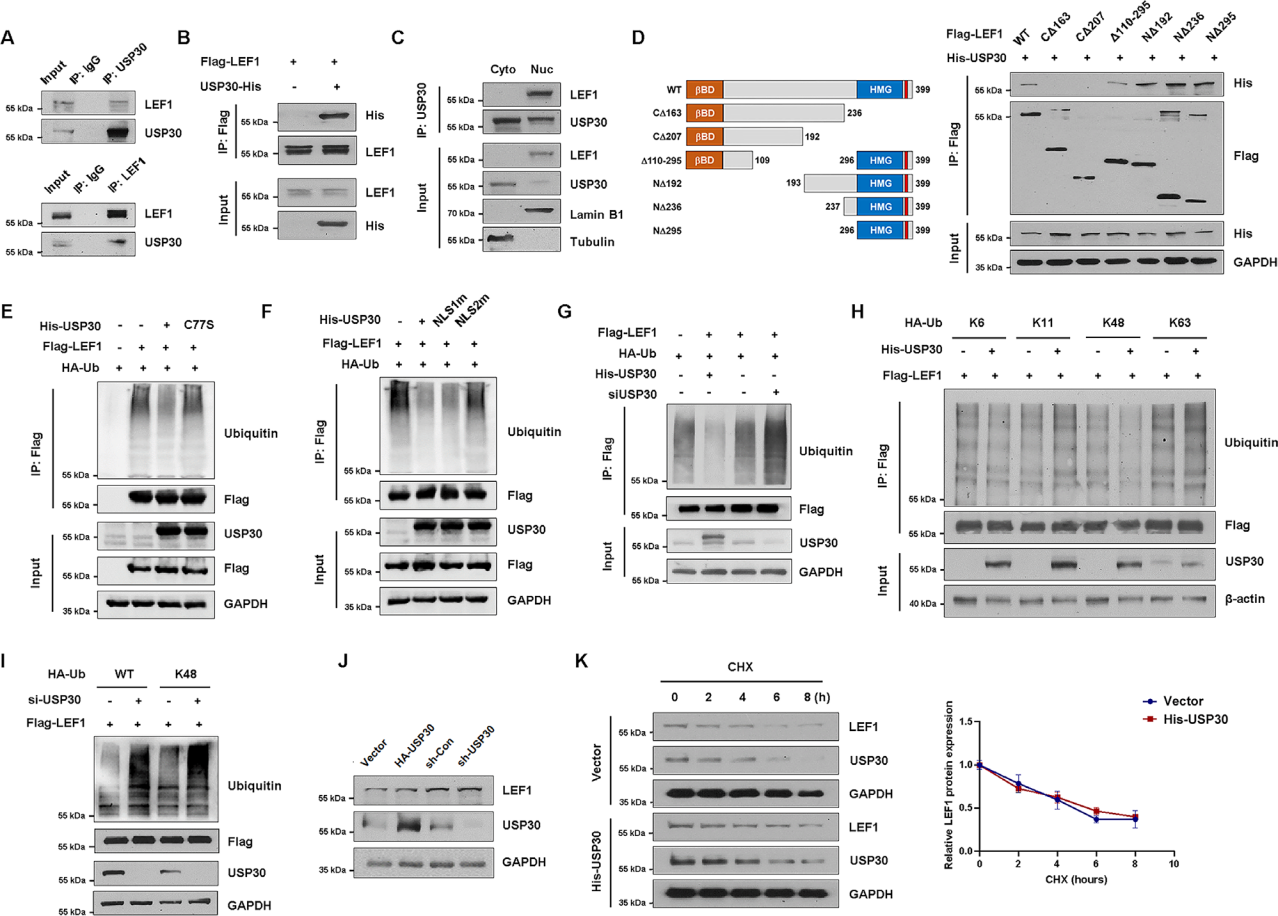
**Transcription factor TCF/LEF1 is a novel substrate of USP30**. (A) Co‐IP assay showed the interaction between endogenous USP30 and nuclear transcription factor TCF/LEF1 in MDA‐MB‐231 cells. (B) Co‐IP assay showed the interaction between exogenous USP30 and LEF1 in MDA‐MB‐231 cells. (C) Co‐IP assay showed the interaction between USP30 and LEF1 in nuclear fractions of MDA‐MB‐231 cells. (D) LEF1 truncation mutants: CΔ163, CΔ207, Δ110‐295, NΔ192, NΔ236, NΔ295. Co‐IP analysis of the interaction between USP30 and LEF1 affected by LEF1 truncation mutations. (E) HEK293 cells were transfected with His‐USP30 or His‐USP30‐C77S, Flag‐LEF1, and HA‐Ub. Cell lysates were immunoprecipitated with anti‐Flag beads and analyzed by immunoblotting with the indicated antibodies. (F) HEK293 cells were transfected with His‐USP30 or His‐USP30 NLSm, Flag‐LEF1, and HA‐Ub. Cell lysates were immunoprecipitated with anti‐Flag beads and immunoblotted with indicated antibodies. (G) HEK293 cells were transfected with His‐USP30, si‐USP30, Flag‐LEF1, and HA‐Ub. Cell lysates were immunoprecipitated with anti‐Flag beads and immunoblotted with indicated antibodies. (H) MAD‐MB‐231 cells were transfected with His‐USAP30, Flag‐LEF1, and HA‐Ub (K6, K11, K48, K63). Cell lysates were immunoprecipitated with anti‐Flag beads and immunoblotted with indicated antibodies. (I) HEK293 cells were transfected with HA‐Ub, HA‐Ub K48, Flag‐LEF1, and si‐USP30. Cell lysates were immunoprecipitated with anti‐Flag beads and immunoblotted with indicated antibodies. (J) Western blot analysis of LEF1 expression in MDA‐MB‐231 cells transfected with HA‐USP30 or sh‐USP30. (K) CHX chase assay of LEF1 and USP30 protein levels in MDA‐MB‐231 cells with or without His‐USP30 overexpression. Band intensities were quantified and presented relative to the 0‐h control (set to 1) based on three independent experiments.

Immunoprecipitation and mass spectrometry analyses identified multiple potential nuclear interaction partners of USP30 (Figure S6A). Co‐IP assays demonstrated a specific interaction between USP30 and the transcription factor TCF/LEF1 in both HEK293 and MDA‐MB‐231 cells (Figure 4A,B and Figure S6B). Importantly, this interaction was exclusively detected in nuclear fractions (Figure 4C). The TCF/LEF family of transcription factors includes several conserved domains, such as the N‐terminal β‐catenin binding domain and the C‐terminal HMG DNA‐binding domain. Structure‐function analysis using various LEF1 truncation mutants (CΔ163, CΔ207, Δ110‐295, NΔ192, NΔ236, and NΔ295) revealed that the C‐terminal HMG domain is essential for USP30 binding (Figure 4D).

To investigate whether LEF1 is a bona fide USP30 substrate, we performed comprehensive ubiquitination assays in MG132‐treated cells. Ectopic expression of WT USP30 and the NLS1m mutant significantly reduced LEF1 ubiquitination, whereas catalytic inactive C77S and nuclear‐localization defective NLS2m mutants showed no effect (Figure 4E,F and Figure S6C). Conversely, knockdown of USP30 markedly increased LEF1 ubiquitination (Figure 4G). USP30 is known to preferentially cleave K6‐ and K11‐linked polyubiquitin chains [30]. Mechanistic studies demonstrated that, however, USP30 specifically cleaves K48‐linked polyubiquitination chains from LEF1 in both HEK293T and MDA‐MB‐231 cells (Figure 4H and Figure S6D), with USP30 depletion increasing K48‐linked ubiquitination (Figure 4I). Interestingly, despite K48‐linked polyubiquitin chains typically targeting proteins for proteasomal degradation, cycloheximide (CHX) chase assays revealed USP30‐mediated deubiquitination does not affect LEF1 protein stability (Figure 4J–K and Figure S6E,F). These findings establish LEF1 as a novel USP30 substrate and suggest USP30 regulates LEF1 function through non‐proteolytic K48‐linked deubiquitination.

### 2.5 USP30 Suppresses WNT Signaling by Inhibiting β‐Catenin/LEF1 Transactivation

**FIGURE 5:**
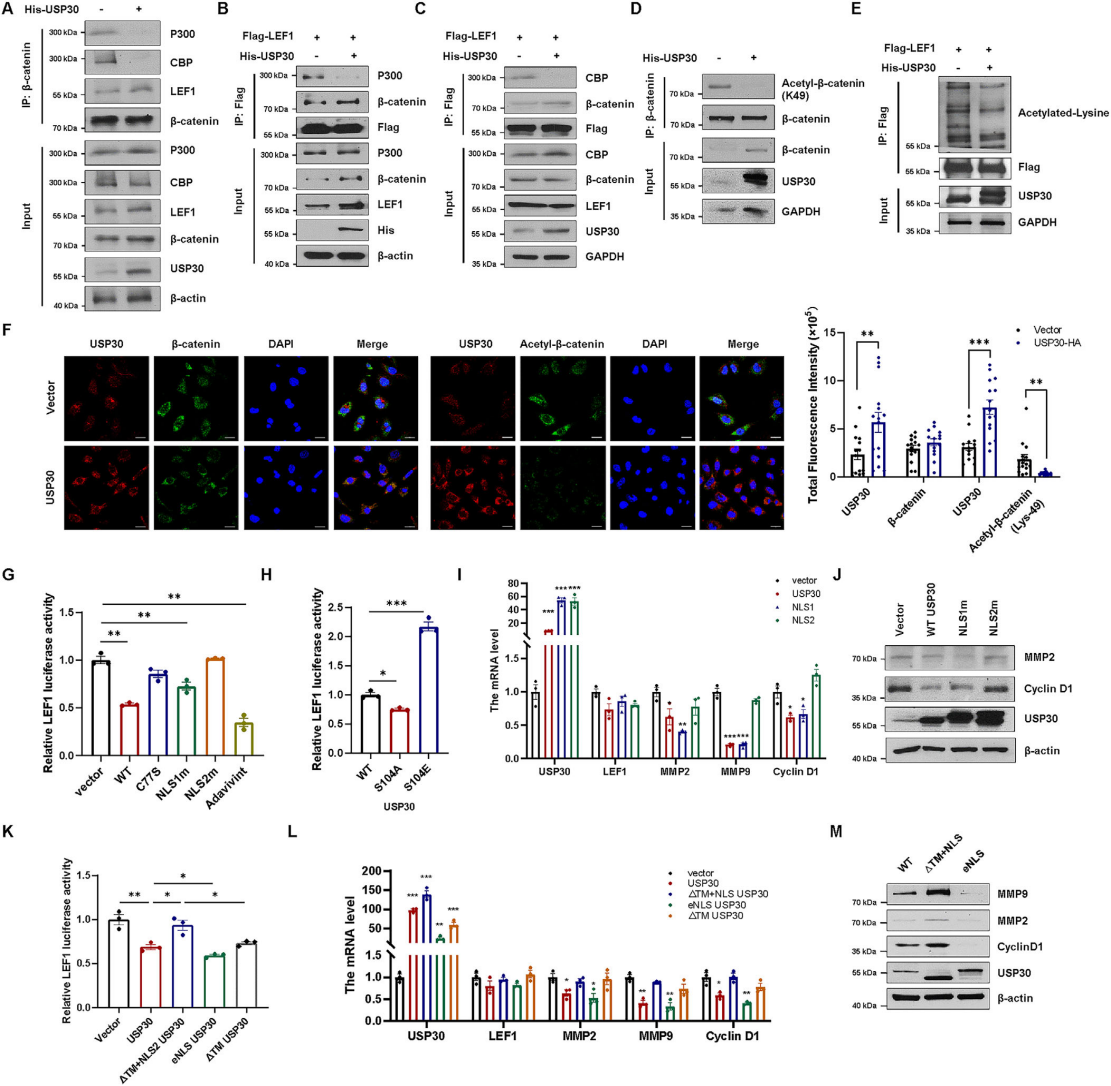
**USP30 inhibits TCF/LEF1 transcription activity by interfering CBP/P300‐mediated acetylation**. (A) Co‐IP analysis of CBP, P300, and LEF1 using anti‐β‐catenin antibody in HEK293 cells with or without His‐USP30 overexpression. (B and C) HEK293 cells were co‐transfected with Flag‐LEF1, His‐USP30, or His‐vector. LEF1 was immunoprecipitated with anti‐Flag beads, and analyzed by immunoblotting with anti‐P300, anti‐CBP, and anti‐β‐catenin. (D) Co‐IP analysis of β‐catenin K49 acetylation using anti‐β‐catenin antibody in HEK293 cells with or without His‐USP30 overexpression. (E) Co‐IP analysis of LEF1 acetylation by anti‐Flag beads in HEK293 cells co‐transfected with Flag‐LEF1, His‐USP30, or His‐vector. (F) Immunofluorescence staining of MDA‐MB‐231 cells transfected with His‐USP30 or His‐vector. Scale bar: 30 µm. The mean fluorescence intensity was quantified by image J. Statistical significance was determined by Student's *t*‐test (^∗^ *p* < 0.05, ^∗∗^ *p* < 0.01, ^∗∗∗^ *p* < 0.001). (G and H) Dual luciferase reporter gene assay was used to assess LEF1 transcriptional activity in MDA‐MB‐231 cells transfected with His‐vector, His‐USP30 or USP30 mutants, or treated with Adavivint (30 nM, 24 h). (I and J) RT‐qPCR and western blot analysis of WNT target genes and proteins in MDA‐MB‐231 cells transfected with His‐vector, His‐USP30, or USP30 NLS mutants. (K) Dual luciferase reporter gene assay was used to assess LEF1 transcriptional activity in MDA‐MB‐231 cells transfected with His‐vector, His‐USP30, or USP30 truncation mutants. (L and M) RT‐qPCR and western blot analysis of WNT target genes and proteins in MDA‐MB‐231 cells transfected with His‐vector, His‐USP30, or USP30 truncation mutants. Data are presented as mean ± SD from three independent experiments. Statistical significance was determined by one‐way ANOVA (∗*p* < 0.05, ∗∗*p* < 0.01, ∗∗∗*p* < 0.001).

Ubiquitination not only regulates protein degradation but also plays a crucial role in various cellular processes, including protein–protein interactions, subcellular localization, signal transduction, and transcriptional regulation [31]. In the canonical WNT signaling pathway, nucleus β‐catenin acts as a molecular scaffold for TCF/LEF1, recruiting co‐activators including the histone acetyltransferases CBP/P300, B‐cell lymphoma (BCL9), and Pygopus (PYGO) to drive expression of WNT target genes [32, 33]. Co‐IP experiments demonstrated that USP30 does not disrupt β‐catenin‐LEF1 interaction or affect the recruitment of BCL9/PYGO to β‐catenin; it specifically prevents CBP/P300 association with the complex (Figure 5A–C and Figure S6G). Additionally, USP30 significantly reduced the acetylation levels of β‐catenin and LEF1 (Figure 5D,E), confirmed by immunofluorescence showing decreased acetyl‐β‐catenin (Lys 49) without affecting total β‐catenin (Figure 5F).

To assess the impact of USP30 on LEF1 transcriptional activity, we conducted TCF/LEF1 luciferase reporter assays in MDA‐MB‐231 cells. Overexpression of WT USP30 significantly reduced LEF1 luciferase activity. Importantly, this suppressive effect was abolished in both the nuclear localization‐deficient NLS2m mutant and catalytic inactive C77S mutant (Figure 5G). In contrast, the nuclear‐enriched S104A mutant exhibited a more pronounced reduction in LEF1 activity compared to WT USP30 (Figure 5H). Quantitative RT‐PCR and western blot analyses further confirmed that WT USP30 downregulated the expression of WNT target genes (Figure 5I,J and Figure S6H). Further characterization of subcellular localization mutants revealed that the ΔTM+NLS mutant failed to alter LEF1 transcriptional activity or WNT target gene expression, but the additional NLS signal potentiated USP30's inhibitory effects on LEF1 transcriptional activity beyond WT levels (Figure 5K–M). These findings underscore the importance of USP30's nuclear localization in modulating WNT/β‐catenin/LEF1 signaling.

### 2.6 USP30‐Mediated LEF1 K379/382 Deubiquitination Inhibits Cancer Stemness and Chemoresistance

**FIGURE 6:**
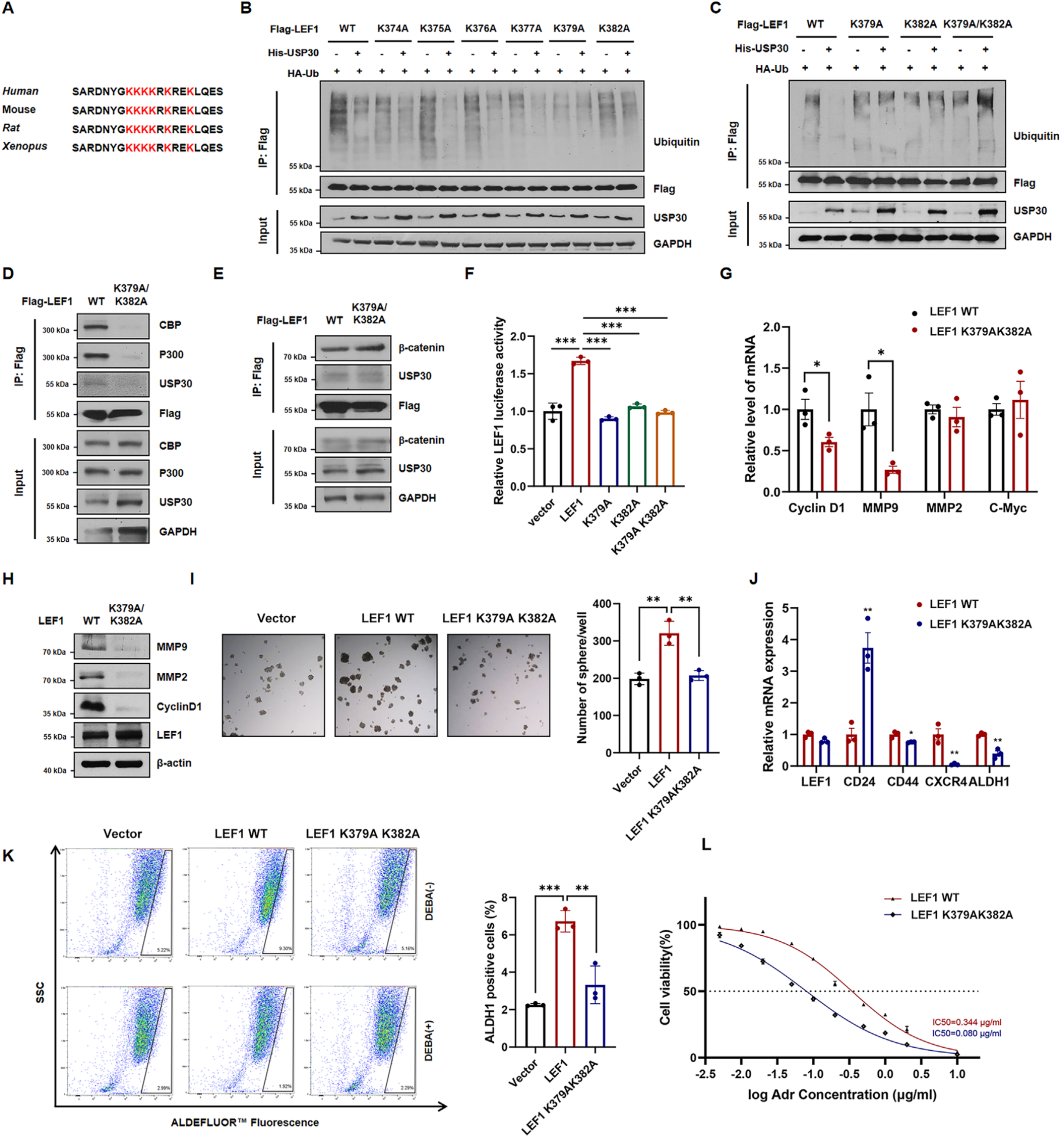
**USP30‐mediated LEF1 K379/382 deubiquitination prevents LEF1 transactivation**. (A) Predicted ubiquitination sites with high confidence scores in the C‐terminal HMG domain of LEF1. (B) Six lysine residues (K374, K375, K376, K377, K379, and K382) were mutated to alanine A. HEK293 cells were transfected with plasmids as indicated. The lysates were immunoprecipitated with anti‐Flag beads and immunoblotted with anti‐ubiquitin to reveal the ubiquitination levels. (C) HEK293 cells were co‐transfected with His‐USP30 or His‐vector, HA‐Ub, and Flag‐LEF1, Flag‐LEF1‐K379A, Flag‐LEF1‐K382A, or Flag‐LEF1 K379A/K382A. The lysates were immunoprecipitated with anti‐Flag beads and immunoblotted with anti‐ubiquitin to reveal the ubiquitination levels. (D and E) HEK293 cells were transfected with Flag‐LEF1 or Flag‐LEF1‐K379A/K382A. The lysates were immunoprecipitated with anti‐Flag beads and immunoblotted with anti‐CBP, anti‐P300, anti‐β‐catenin, and anti‐USP30. (F) Dual luciferase reporter gene assay was used to assess LEF1 transcriptional activity in MDA‐MB‐231 cells transfected with Flag‐vector, Flag‐LEF1, or LEF1 K379 K382 mutants. Data are presented as mean ± SD from three independent experiments. Statistical significance was determined by one‐way ANOVA (∗*p* < 0.05, ∗∗*p* < 0.01, ∗∗∗*p* < 0.001). (G) RT‐qPCR analysis of WNT target genes in MDA‐MB‐231 cells transfected with Flag‐LEF1 or Flag‐LEF1‐K379A/K382A. Statistical significance was determined by Student's *t*‐test. (H) Western blot analysis of WNT target proteins in MDA‐MB‐231 cells transfected with Flag‐LEF1 or Flag‐LEF1‐K379A/K382A. (I) Sphere formation assay in MDA‐MB‐231 cells transfected with Flag‐LEF1 or Flag‐LEF1K379A/K382A. Statistical significance was determined by one‐way ANOVA. (J) RT‐qPCR analysis of CSC‐like behavior‐related genes in MDA‐MB‐231 cells transfected with Flag‐LEF1 or Flag‐LEF1 K379A/K382A. Statistical significance was determined by Student's *t*‐test. (K) Flow cytometry analysis of ALDH activity in MDA‐MB‐231 spheres transfected with Flag‐Vector Flag‐LEF1 or Flag‐LEF1‐K379A/K382A. Statistical significance was determined by one‐way ANOVA. (L) MDA‐MB‐231 cells with LEF1 or LEF1 K379A K382A overexpression were exposed to different concentrations of Adriamycin for 72 h, and the cell viability was determined by CCK‐8. Data were normalized to the wide‐type control (set as 100%) and are presented as mean ± SD from three independent experiments.

To identify the specific LEF1 residues that are deubiquitinated by USP30, we performed bioinformatic analysis using protein ubiquitination sites prediction algorithms (BDM‐PUB, UbPred, and UbiSite). The C‐terminal HMG domain of LEF1 displayed multiple putative ubiquitination sites with high confidence scores, with notable evolutionary conservation across human, mouse, rat, and xenopus species (Figure 6A). We then systematically mutated six candidate lysine residues (K374, K375, K376, K377, K379, and K382) to either arginine (R) or alanine (A) for functional validation. Overexpression of USP30 effectively reduced ubiquitinated of WT LEF1, as well as single mutants (K374A, K375A, K376A, and K377A), while showing no effect on K379A, K382A, or the K379A/382A double mutant. These results indicate that USP30 specifically cleaves K48‐linked polyubiquitin chains at K379 and K382 residues (Figure 6B,C and Figure S7A,B).

Subsequently, we examined how USP30‐mediated deubiquitination affects β‐catenin/LEF1 complex function. Co‐IP experiments revealed both WT and K379A/K382A mutant LEF1 interact with β‐catenin, but the mutant showed impaired recruitment of CBP/P300 to the β‐catenin/LEF1 complex (Figure 6D,E). Consistently, transcriptional reporter assays and expression profiling revealed significantly reduced transcriptional activity and downstream WNT target gene expression in K379A, K382A, and K379A/K382A mutants compared to WT LEF1 (Figure 6F–H and Figure S7C). Functional characterization further demonstrated that these ubiquitination‐deficient mutants lost their capacity to promote CSC‐like properties and chemoresistance in MDA‐MB‐231 cells (Figure 6I–L and Figure S7D).

### 2.7 USP30 Expression in Breast Cancer and Its Association With Hormone Receptor Status

**FIGURE 7:**
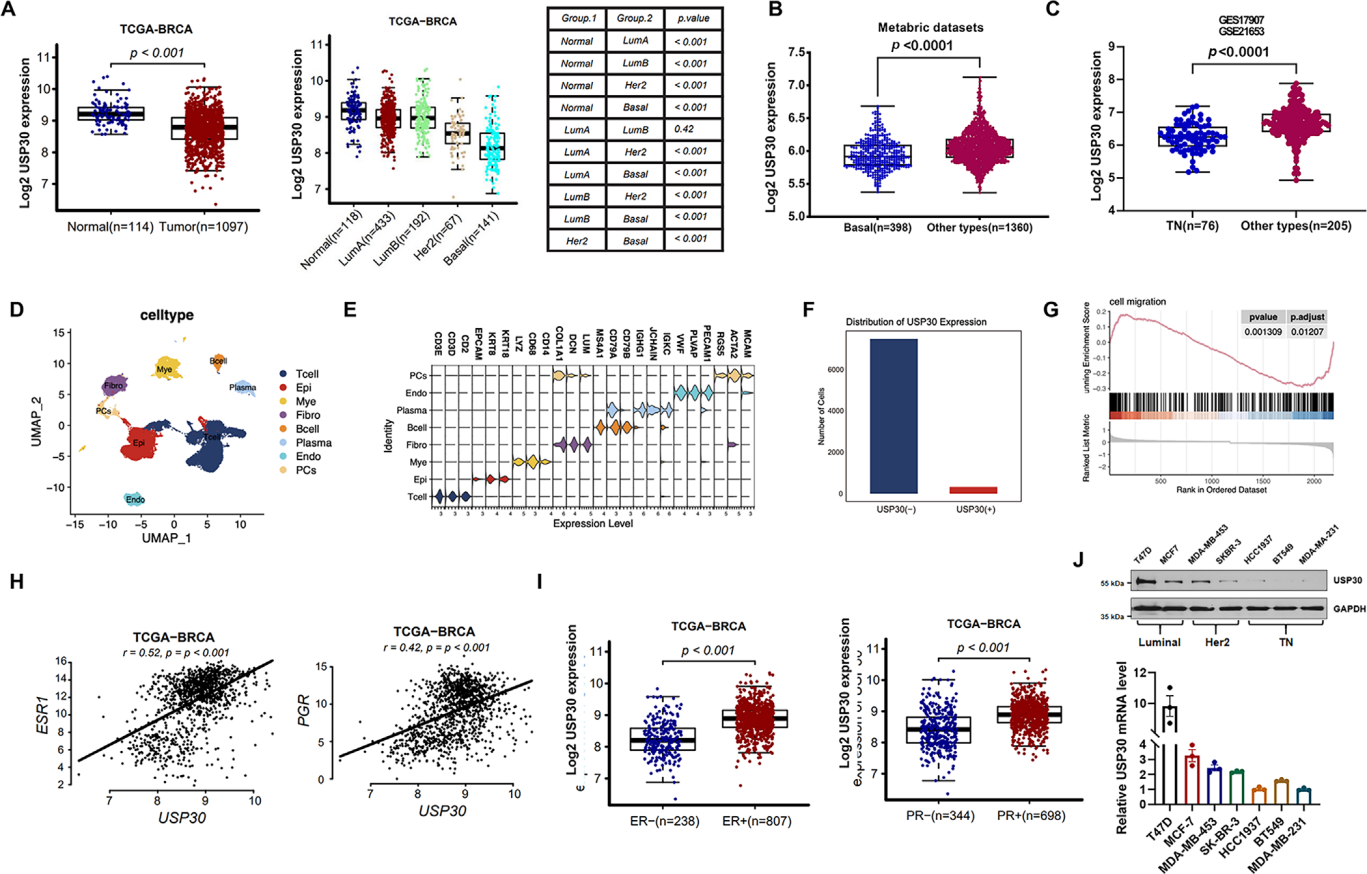
USP30 expression in breast cancer and its association with hormone receptor status. (A) Comparison of USP30 expression between normal tissues (*n* = 114) and breast cancer tissue (*n* = 1097) from the TCGA database. Expression levels were also compared across breast cancer subtypes, including normal breast tissue (*n* = 118), luminal A (*n* = 433), luminal B (*n* = 192), HER2+ (*n* = 67), and basal‐like (*n* = 141) breast cancer. (B) Comparison of USP30 expression between basal‐like breast cancer (*n* = 398) and other types of breast cancer (*n* = 1360) from the METABRIC datasets. (C) Comparison of USP30 expression between triple‐negative breast cancer (TNBC) (*n* = 76) and other types of breast cancer (*n* = 205) from the GEO database. ^***^ *p* < 0.001, by Mann–Whitney *U* test. (D) UMAP plot showing the distribution of eight major cell types, with cells colored by cell type. (E) Violin plots displaying cell type‐specific marker gene expression used for annotation. (F) Bar plot showing the number of USP30‐positive (USP30⁺) and USP30‐negative (USP30^−^) epithelial cells. (G) GSEA reveals enrichment of differentially expressed genes in the EMT pathway. (H) The Spearman correlation analysis of mRNA levels between USP30 and ESR1/pGR in TCGA breast cancer cohorts (*n* = 500). (I) Comparison of USP30 mRNA levels between ER‐negative (*n* = 238) and ER‐positive (*n* = 807) or PR‐negative (*n* = 344) and PR‐positive (*n* = 698) breast cancer tissue from TCGA cohorts. ^***^ *p* < 0.001, by Mann–Whitney *U* test. (J) USP30 expression levels in luminal (MCF‐7 and T47D), Her2‐enriched (MDA‐MB‐453, and SKBR3), and TNBC (HCC1937, BT549, and MDA‐MB‐231) cell lines analyzed by western blotting and RT‐qPCR. Data were normalized and are presented as mean ± SD from three independent experiments.

To evaluate the clinical significance of USP30 in breast cancer, we performed a comprehensive analysis of TCGA, GEO, and METABRIC datasets. The results demonstrated that USP30 transcripts are significantly reduced in breast cancer tissues compared to normal breast tissue, with basal‐like (primarily triple‐negative) specimens showing the most pronounced downregulation among all molecular subtypes (Figure 7A–C and Figure S8A–C). This clinical finding was corroborated by single‐cell RNA‐seq analysis of 32,803 high‐quality cells from 13 breast cancer datasets (E‐MTAB‐8107), where USP30 expression was restricted to a minor epithelial subpopulation (Figure 7D–F and Figure S8D). Differential expression analysis revealed that USP30‐positive epithelial cells exhibited significant downregulation of EMT pathways, especially genes involved in cell adhesion and substrate junction formation, suggesting USP30's potential role in maintaining epithelial differentiation and suppressing malignant progression (Figure 7G and Figure S8E).

Genetic correlation analysis revealed significant positive correlations between USP30 expression and ESR1 (estrogen receptor alpha) and PGR (progesterone receptor) levels in breast cancer samples (Figure 7H). Clinically, ER‐ and PR‐positive breast cancer tissues showed higher USP30 levels than receptor‐negative cases (Figure 7I and Figure S8F,G). Consistent with these findings, TNBC cell lines (HCC1937, BT549, and MDA‐MB‐231) showed markedly reduced USP30 expression relative to luminal (MCF‐7 and T47D) and Her2‐enriched (MDA‐MB‐453 and SKBR3) cell lines (Figure 7J). These results establish a robust association between USP30 expression and hormone receptor status across both clinical samples and experimental models.

### 2.8 Nuclear USP30 Suppresses Lung Metastasis by Deubiquitinating LEF1

**FIGURE 8:**
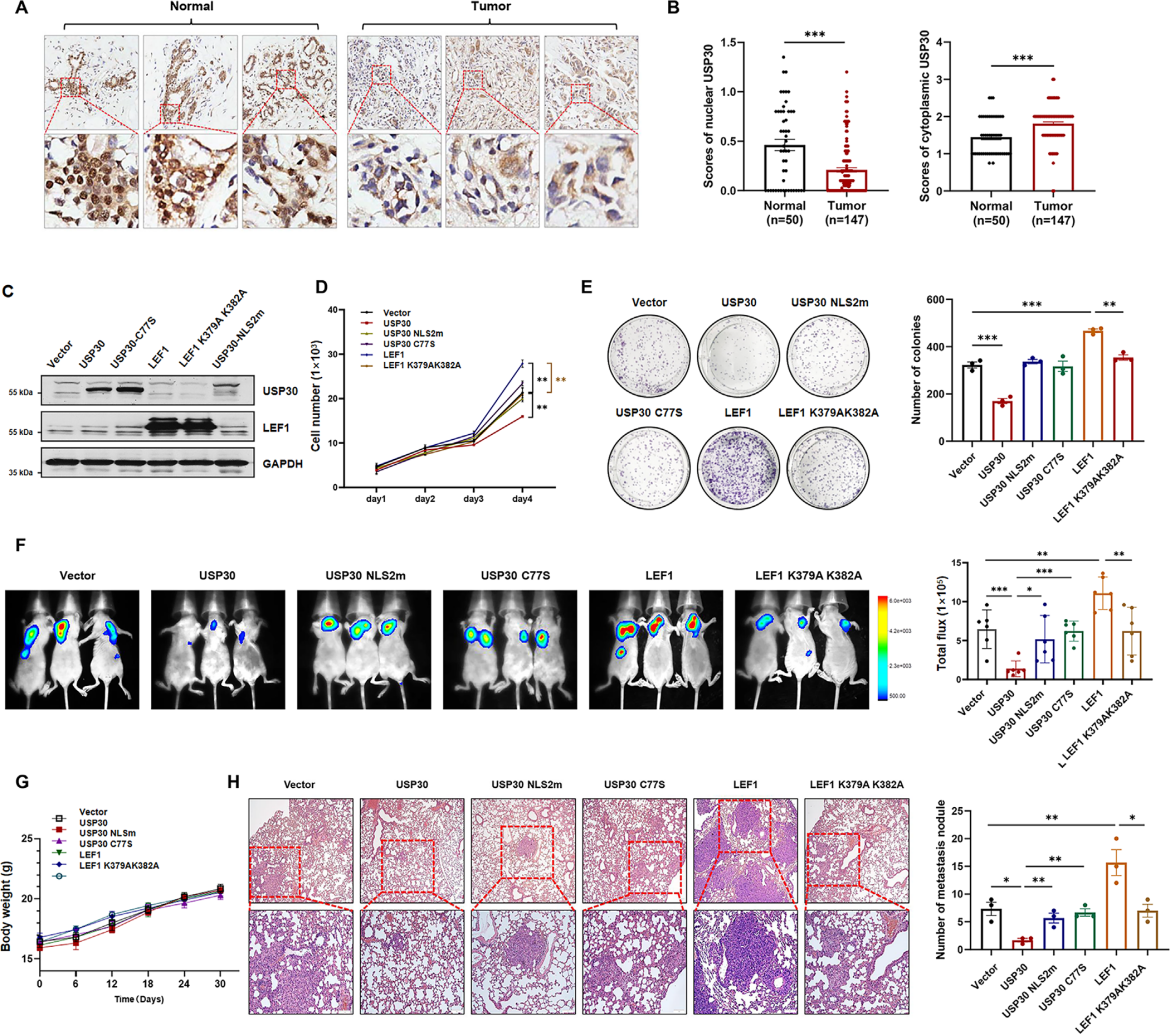
**Nuclear USP30 suppresses lung metastasis by deubiquitinating LEF1**. (A and B) Immunohistochemical staining of USP30 in a tissue microarray containing 147 breast carcinoma samples and 50 normal mammary tissues. The scores of USP30 were determined by evaluating the extent and intensity of immunopositivity. Statistical significance was determined by Student's *t*‐test. (C) Western blot analysis of USP30 and LEF1 expression in MDA‐MB‐231 cells infected with lentiviruses carrying Vector‐luc, USP30‐luc, USP30‐C77S‐luc, LEF1‐luc, LEF1‐K379AK382A‐luc, or USP30‐NLS2 m‐luc. (D) Cell viability of MDA‐MB‐231 cells infected with lentiviruses carrying Vector‐luc, USP30‐luc, USP30‐C77S‐luc, LEF1‐luc, LEF1‐K379AK382A‐luc, or USP30‐NLS2‐luc analyzed by CCK‐8. (E) Representative images and quantification of colony formation assay in MDA‐MB‐231 cells infected with lentiviruses carrying Vector‐luc, USP30‐luc, USP30‐C77S‐luc, LEF1‐luc, LEF1‐K379AK382A‐luc, or USP30‐NLS2m‐luc. Data are presented as mean ± SD, and statistical significance was determined by one‐way ANOVA (∗*p* < 0.05, ∗∗*p* < 0.01, ∗∗∗*p* < 0.001). (F) Representative bioluminescent images and quantification of lung metastasis in mice injected with MDA‐MB‐231‐Luc cells (*n* = 6 mice per group). Data are presented as mean ± SD, and statistical significance was determined by one‐way ANOVA (∗*p* < 0.05, ∗∗*p* < 0.01, ∗∗∗*p* < 0.001). (G) Body weight monitoring of six groups of mice (*n* = 6 mice per group). Data are presented as mean ± SD. No significant differences were found among groups by two‐way repeated measures ANOVA. (H) Representative H&E staining images of lung tissues from six groups of mice (left), and quantification of the number of lung metastases (right). Scale bar, 100 µm. Data are presented as mean ± SD, and statistical significance was determined by one‐way ANOVA (∗*p* < 0.05, ∗∗*p* < 0.01, ∗∗∗*p* < 0.001).

Finally, we performed immunohistochemical analysis on breast cancer tissue microarrays. Quantitative evaluation demonstrated that nuclear USP30 levels were significantly reduced in tumor tissues compared to adjacent normal breast tissues (Figure 8A,B). Considering the established function of nuclear USP30 in inhibiting WNT signaling and cancer stemness characteristics, we hypothesized that enhancing nuclear USP30 expression could impede breast cancer metastasis. We generated stable MDA‐MB‐231 cell lines expressing USP30 WT, the NLS2m mutant, the C77S mutant, LEF1 WT, and the K379A/K382A mutant, along with firefly luciferase (Figure 8C). Cell proliferation assays demonstrated that overexpression of USP30 WT inhibited cell proliferation and colony formation compared to the vector control, whereas both the NLS2m and C77S mutants lost these inhibitory effects. Conversely, overexpression of LEF1 WT significantly enhanced cell proliferation and colony formation, while the K379A/K382 mutant had no discernible impact (Figure 8D,E).

Subsequently, we investigated the role of nuclear USP30 in cancer progression and metastasis in vivo. MDA‐MB‐231 cells stably expressing the above‐mentioned constructs were intravenously injected into the tail vein of female BALB/c nude mice. The growth and seeding of metastatic cells in the lungs were non‐invasively monitored using quantitative bioluminescent imaging. By day 9 post injection, significant lung seeding was detected in mice receiving cells with the vector, USP30 mutants, or LEF1. This seeding effect persisted for 20 days. During this period, USP30 significantly suppressed lung metastasis, whereas the C77S and NLS2m mutants had similar effects to the vector control. Conversely, overexpression of LEF1 markedly enhanced lung cancer metastasis, while the K379A/K382 mutant had no significant effect (Figure 8F,G and Figure S9A,B). At 30 days after injection, mice were sacrificed, and lung metastases were quantified. USP30 notably reduced the formation of lung metastatic loci compared to the vector control. However, the anti‐metastatic effect of USP30 was nullified by the NLS2m and C77S mutants. In contrast, LEF1 overexpression significantly increased the number of lung metastatic nodules, whereas the K379A/K382A mutant lost its pro‐metastatic potential (Figure 8H). Similar results were observed using the 4T1 TNBC murine cell line (Figure S9C). These findings indicate that nuclear‐localized USP30 function as a tumor suppressor by modulating the ubiquitination status of TCF/LEF1.

## 3 Discussion

Mitophagy is a vital quality control mechanism that removes dysfunctional or excess mitochondria to maintain mitochondrial homeostasis. Dysregulation of mitophagy regulators, such as PINK1, Parkin, BNIP3, NIX, and FUNDC1, have been implicated in cancer progression [34, 35, 36, 37]. As the only known active DUB constitutively anchored in the outer mitochondrial and peroxisome membranes, USP30 antagonizes PINK1/Parkin‐mediated mitophagy during mitochondrial depolarization [38, 39]. Previous studies have demonstrated that dysregulation of USP30 in mice with high‐fat diet promotes lipogenesis and liver cancer progression through IKKβ–USP30–ACLY axis [27]. USP30 has also been implicated in breast cancer progression by stabilizing Snail [28]. Our study unveils a novel tumor‐suppressive mechanism of nuclear USP30 in breast cancer cells through mitochondrial retrograding signaling. We demonstrate that nuclear USP30 binds to and deubiquitinates TCF/LEF1, thereby preventing P300/CBP recruitment to the β‐catenin/LEF1 complex and reducing TCF/LEF1 acetylation and transcriptional activity.

The WNT signaling pathway is regulated by PTMs, including ubiquitination and deubiquitination of key factors such as β‐catenin, AXIN, DVL, and GSK3 [40, 41, 42, 43, 44, 45]. TCF/LEF1, a key transcription factor in canonical Wnt/β‐catenin signaling pathway, is overexpressed in HER‐2 negative breast cancers [46, 47] and has been implicated in brain metastasis through modulation of glutathione metabolism and ROS resistance [48]. Our findings establish USP30 as a novel regulator that removes K48‐linked polyubiquitin chains from TCF/LEF1 at K379 and K382. Importantly, USP30‐mediated deubiquitination prevents P300/CBP recruitment to the β‐catenin/LEF1 transcriptional complex without affecting LEF1 protein stability. This contrasts with PJA2‐mediated ubiquitination, which directly targets LEF1 for proteasomal degradation [49], suggesting distinct functional consequences of ubiquitination at different sites or chain linkages. Notably, OTUD7B regulates LEF1/β‐catenin interaction via nuclear localization rather than ubiquitination [50], highlighting spatial specificity of deubiquitination enzymes in WNT regulation. Therefore, our results provide new insights into the molecular mechanisms underlying WNT signaling dysregulation in breast cancer, particularly the role of non‐proteolytic ubiquitination in transcriptional complex assembly.

The coordination between mitochondria and the nucleus is crucial for cellular homeostasis and stress responses. While anterograde signaling regulates mitochondrial function, retrograde signaling affect nuclear gene expression, metabolic reprogramming, and cancer stem cell (CSC) behavior [15, 51, 52]. Targeting this crosstalk holds significant potential for eradicating CSCs and providing therapeutic benefits for cancer patients [53]. Although USP30 is primarily localized to the mitochondrial outer membrane, our findings reveal its dual localization in both mitochondria and the nucleus. Phosphorylation can either enhance or inhibit nuclear import [54]. We demonstrate that nutrient deprivation and mTOR inhibitors promote USP30 nuclear translocation, with phosphorylation at serine 104, a mTORC1‐dependent modification predominantly detected in cytoplasmic USP30. The S104A mutation exhibits enhanced nuclear localization and shows more potent inhibitory effect on CSCs‐like properties, further supporting the tumor‐suppressive role of nuclear USP30.

Recent studies highlight extensive crosstalk between mitochondria and WNT signaling [55]. WNT signaling plays a significant role in modulating various aspects of mitochondrial biology, including biogenesis, metabolism, fission, and fusion. Conversely, mitochondrial retrograde signaling directly regulates WNT pathway, which is crucial for maintaining CSCs and promoting cancer progression [56, 57, 58, 59]. Exploiting mitochondrial/WNT crosstalk may offer a novel therapeutic strategy for cancer treatment. Through compartment‐specific USP30 mutants, we establish a functional dichotomy: mitochondrial USP30 regulates mitophagy, while nuclear USP30 inhibits WNT signaling to exert anti‐tumor effects. Notably, comprehensive gene expression and breast cancer tissue microarray analyses reveal that USP30 is significantly downregulated in TNBC and CSCs, with reduced nuclear localization in tumors, suggesting clinical relevance.

While our findings establish the mTORC1‐USP30‐LEF1 axis as a critical regulator of cancer stemness, several important questions remain open for investigation. First, the biological relevance of USP30 relocalization in spontaneous metastasis models and its interplay with immune microenvironment components require further exploration. Second, the upstream signaling that connects nutrient deprivation sensors to mTORC1‐mediated phosphorylation of USP30 at S104 needs to be fully elucidated. Third, whether additional nuclear substrates beyond LEF1 contribute to USP30's tumor suppressive functions warrants systematic identification. Finally, the clinical significance of nuclear USP30 localization as a potential biomarker awaits validation in large‐scale patient cohorts to determine its prognostic and predictive utility. Addressing these questions will advance our understanding of mitochondria‐nuclear communication in cancer.

In summary, this study demonstrates that USP30 exhibits dual mitochondrial and nuclear localization, with nutrient stress dynamically promoting its nuclear translocation via mTORC1‐dependent phosphorylation at Ser104. Within the nucleus, USP30 acts as a tumor suppressor by deubiquitinating LEF1 at K379/K382, disrupting the β‐catenin/LEF1 transcriptional complex and inhibiting cancer stemness, chemoresistance, and metastasis in TNBC. This study resolves a key knowledge gap regarding USP30's nuclear functions and provides mechanistic insight into mitochondrial‐nuclear communication in cancer. Targeting the dynamic relocalization of USP30 from mitochondria to the nucleus may offer novel therapeutic strategies for combating breast cancer metastasis.

## 4 Materials and Methods

### 4.1 Cell Culture

The following cell lines were sourced from the American Type Culture Collection (Manassas, VA, USA): MDA‐MB‐231, MCF7, HEK293, T47D, MDA‐MB‐453, SKBR‐3, HCC1937, 4T1, and BT549. MDA‐MB‐231, MCF7, SKBR‐3, and HEK293 cells were cultured in high‐glucose Dulbecco's modified Eagle medium (DMEM, Gibco, USA) supplemented with 10% fetal bovine serum (FBS), at 37°C in a 5% CO_2_ atmosphere. T47D, HCC1937, and BT549 cells were maintained in RPMI‐1640 medium (Gibco, USA) with 10% FBS under the same conditions. MDA‐MB‐453 cells were grown in L‐15 medium (Gibco, USA) with 10% FBS at 37°C. 4T1 cells were cultured in a 1:1 mixture of DMEM and F12 medium (Gibco, USA), also supplemented with 10% FBS, at 37°C in 5% CO_2_.

### 4.2 Nutrient Starvation

For serum starvation, cells in the experimental group were washed twice with phosphate‐buffered saline (PBS) to remove residual serum components and incubated in serum‐free medium to induce serum deprivation, while control cells were maintained in complete medium supplemented with 10% FBS. For glucose starvation, experimental cells were transferred to low‐glucose medium (1.0 g/L glucose DMEM) containing 10% FBS to restrict glucose availability, whereas control cells were cultured in complete high‐glucose medium (4.5 g/L glucose DMEM) supplemented with 10% FBS to maintain normal glucose levels. All cells were incubated under standard conditions (37°C, 5% CO_2_) for the designated treatment duration, and starvation experiments were terminated at predetermined time points for subsequent analysis.

### 4.3 Transfection and Construction of Stable Cell Lines

The pcDNA3.1‐USP30, pcDNA3.1‐LEF1, pcDNA3.1‐Ub, and their corresponding mutant and truncated plasmids were generated by Youbao (Hunan, China). The pLent‐EF1a‐P2A‐Luciferase‐CMV‐copGFP‐P2A‐Puro‐USP30, LEF1, and their respective mutant lentivirus plasmids were constructed by Weizhen Biosciences (Shandong, China). The authenticity of all constructs was verified by DNA sequencing. The design and synthesis of siRNA targeting USP30 were performed by Gene Pharma (Shanghai, China). Lentivirus vectors containing short hairpin RNA against USP30 (shUSP30) and negative control were acquired from GeneChem Company (Shanghai, China). Plasmid or siRNA transfections were carried out using Jetprime (Polyplus, #101000046) transfection reagent. Lentivirus transfections followed the protocol provided by Gene Pharma (Shanghai, China), and lentivirus‐infected cell lines were selected and maintained in medium containing 2 mg/mL puromycin (Beyotime, ST551) to generate stable cell lines. The siRNA sequences are listed in Table S1.

### 4.4 Sphere Formation Assay

Cells were seeded into ultra‐low attachment six‐well plates (Corning, USA) at a density of 10^4^ cells/mL and cultured in DMEM/F12 supplemented with 20 ng/mL bFGF (MesChemExpress, #HY‐P7331), 20 ng/mL EGF (MesChemExpress, #HY‐P700051AF), and 2% B27 (Invitrogen, #17504044) for 7–9 days, with medium changes every 3 days.

### 4.5 Western Blotting

Cells were washed with cold PBS, then harvested and treated with NP‐40 buffer (Beyotime, #P0013F). Protein concentrations were measured using the Bradford protein assay kit (Beyotime, #P0006C). Forty micrograms of protein lysates were separated by 7.5%–12.5% SDS‐PAGE (Epizyme, Shanghai, China), transferred to a nitrocellulose membrane (Pall Corporation, #66485), and blocked with 5% non‐fat milk in TBST for 1 h at room temperature. Membranes were incubated with primary antibodies overnight at 4°C, washed with TBST, and then incubated with goat anti‐rabbit/mouse secondary antibodies (1:5000, SeraCare, USA) for 1 h at room temperature. After washing with TBST, protein band images were captured. The antibodies used are listed in Table S2.

### 4.6 Co‐immunoprecipitation

Whole cellular extracts were prepared using NP40 buffer with protease inhibitors. Protein A/G Magnetic Beads (Bimake) conjugated with antibodies against the target protein were added to 1 mg of whole cellular extracts and incubated overnight at 4°C with rotation. After washing three times with PBST, the bound components were eluted by boiling with SDS‐PAGE loading buffer and then analyzed by western blot.

### 4.7 Immunofluorescence

Cells were grown to 50% confluence on 14 mm circular cover glasses at 37°C. After three washes with PBS, they were fixed with 4% paraformaldehyde for 15 min and then washed three times with PBS. Permeabilization was done with 0.25% Triton X‐100 for 15 min, followed by three PBS washes. Cells were blocked with 3% BSA for 1 h at room temperature and then incubated with primary antibodies overnight at 4°C. After three PBS washes, they were incubated with Alexa Fluor 488/555‐conjugated secondary antibody (CST, 1:200) for 1 h. Cells were then washed three times with PBS and stained with DAPI (Beytime, #P0131‐25 mL). Images were captured using a Nikon Eclipse Ti Confocal Laser Fluorescence Microscope.

### 4.8 RNA Extraction and RT‐qPCR

Total RNA was extracted from cultured cells using Trizol reagent (Thermo Fisher, #15596026). One microgram of total RNA was reverse‐transcribed with TransScript All‐in‐One First‐Strand cDNA Synthesis SuperMix (Trans, #AE341‐03). mRNA expression levels were quantified by real‐time PCR using PerfectStart Green qPCR SuperMix (Trans, #AQ601) on a qTOWER 2.0 (Analytik Jena AG, Germany). The relative expression levels of mRNA were evaluated using the 2^−ΔΔCt^ method. Primer sequences are provided in Table S1.

### 4.9 Luciferase Reporter Assay

The luciferase activity assay involved transfecting Myc‐TA‐luc plasmid with TCF/LEF1 binding sites and co‐transfecting renilla luciferase encoding plasmids (pRL‐TK) as an internal control. At 48 h post‐transfection, luciferase activities were measured using the Dual‐Luciferase Reporter Assay System (Promega, USA) following the manufacturer's protocol.

### 4.10 FACS Analysis

ALDH activity was measured using the ALDEFLUOR kit (Stemcell Technologies, #01700). Cell spheres were resuspended in ALDEFLUOR Assay Buffer and adjusted to 1 × 10^6^ cells/mL. Five microliters of activated ALDEFLUOR Reagent was added to 1 mL of cell sample and mixed to prepare the test sample. An equal volume of the mixed suspension was separated as the control sample, to which ALDEFLUOR DEAB Reagent was added and mixed immediately. The samples were incubated at 37°C in the dark for 30–60 min, then centrifuged at 250 × *g* for 5 min. The supernatant was discarded, and the cell pellets were resuspended in 500 µL ALDEFLUOR Assay Buffer and kept on ice. The samples were filtered through a 400‐mesh sieve and analyzed using a Cytoflex Flow Cytometer (Beckman, USA).

### 4.11 Subcellular Fractionation

The cytoplasmic and nuclear fractions were separated using the Nuclear and Cytoplasmic Protein Extraction Kit (Beyotime, #P0028) according to the manufacturer's protocol. The cytoplasmic, mitochondrial, and nuclear fractions were separated using the Cell Fractionation Kit (Abcam, #ab109719) according to the manufacturer's protocol.

### 4.12 In Vivo Xenograft Experiments

Female BALB/c nude mice (3–5 weeks old) were purchased from Guangdong Medical Laboratory Animal Center (Guangdong, China). All procedures involving mice and experimental protocols were approved by the Institutional Animal Care and Use Committee of Laboratory Animal Center of Peking University Shenzhen Graduate School and the Bioethics Committee of Tsinghua University Shenzhen International Graduate School (Ethics issue (2022) No. 25). The female BALB/C nude mice were maintained in the mouse‐specific pathogen‐free (SPF) facility for 1 week before injection to allow the mice to adjust to the new environment. Forty BALB/C nude mice were randomly divided into six groups. MDA‐MB‐231 cells (1 × 10^6^) or 4TI cells (1 × 10^6^) and their derivatives were suspended in 100 µL of PBS and then injected into the tail vein of nude mice. The tumor cell metastatic pathways in mice were monitored using the IVIS Spectrum (Perkin Elmer) or Bruker MI SE in vivo imaging system. Successful establishment of the mouse lung metastasis models was demonstrated if lung metastasis appeared in the control group. Mice were killed on day 30 (MDA‐MB‐231 cells) or day 12 (4T1) post‐injection.

### 4.13 Tissue Microarray and Immunohistochemistry (IHC) Scoring

A total of 147 breast invasive ductal carcinoma tissue samples, along with their overall survival data, were obtained from Shanghai Outdo Biotech Co., Ltd (Shanghai, China). Immunohistochemical staining was performed on these patient tissues using anti‐USP30 antibodies at a dilution of 1:250. The IHC score was determined by combining the intensity of staining (0 = no staining, 1 = weak staining, 2 = moderate staining, 3 = strong staining) with the proportion of stained cells (0 = 0%, 1 = 1%–25%, 2 = 26%–50%, 3 = 51%–75%, 4 = 76%–100%).

### 4.14 Statistical Analysis

Statistical analysis was conducted using GraphPad Prism version 8.0. Data are presented as mean ± SD. Differences between two groups were assessed using Student's *t*‐test, while one‐way ANOVA was employed for comparisons among multiple groups. The results are based on at least three independent experiments performed under identical conditions. Statistical significance was defined as *p < 0.05, **p < 0.01, and ***p < 0.001.

## Author Contributions

Conceptualization, design of the study, funding acquisition, supervision, and writing: N.H.X. Methodology, investigation, validation, formal analysis, and writing: X.L.L. and H.W.Z. Methodology, resources, and formal analysis: J.L., C.L., Z.J.Y., J.C., L.X., Y.P.J., R.N.W, H.L.Z, Y.T.L., H.T.Y., and T.G. Conceptualization, project administration, and writing review and editing: W.D.X. and Y.O.Z. All authors have read and approved the final manuscript.

## Ethics Statement

Animal experiments in this study were reviewed and approved by the Institutional Animal Care and Use Committee of Laboratory Animal Center of Peking University Shenzhen Graduate School and the Bioethics Committee of Tsinghua University Shenzhen International Graduate School (Ethics issue (2022) No. 25).

## Conflicts of Interest

The authors declare no conflicts of interest.

## Supporting information




**Figure S1**: Phosphorylation at serine 104 regulates the nuclear translocation of USP30. (A) Western blot analysis of USP30 in cytoplasmic (Cyto) and nuclear (Nuc) fractions of HEK293, HepG2, and HeLa cells. Ratios of cytoplasmic‐to‐nuclear USP30 were quantified using image J. (B) Subcellular fractionation and immunoblotting were performed, and Image J was used to analyze the relative protein expression of USP30 in nuclei and mitochondria at different time points of serum deprivation. Data presented are derived from three independent experiments. (C) Immunofluorescence staining and quantification of mean fluorescence intensity of USP30 in MDA‐MB‐231 cells after glucose restriction (4 h) and serum deprivation (4 h). Scale bar, 15 µm. Data are presented as mean ± SD, statistical significance was determined by one‐way ANOVA (^∗∗∗^p < 0.001).(C) Representative USP30‐interacting proteins identified by mass spectrometry. (D‐E) Western blot analysis of USP30 subcellular localization in MDA‐MB‐231 cells treated with D4476 (CKI inhibitor: 20 µM, 12 h), Silmitasertib (CKII inhibitor: 1 µM, 12 h), H 89 2HCL (PKA inhibitor: 20 µM, 2 h), and SB202190(FHPI) (MAPK inhibitor: 50 mM, 24 h). (F) Co‐IP assay showing the interaction between endogenous USP30 and mTOR in MDA‐MB‐231 cells. (G) Co‐IP analysis of P‐S/T levels of USP30 in different cellular fractions.
**Figure S2**: Gene co‐expression analysis of USP30 in breast cancer. (A) Gene co‐expression analysis between USP30 and CSC‐like behavior related genes in breast cancer cohorts from the CBioportal database. (B‐F) Gene co‐expression analysis between USP30 and MMP9, MYC, OCT4, ALDH1A3, CD133 in breast cancer cohorts.
**Figure S3**: USP30 inhibits cancer stemness and chemoresistance in TNBC cells. (A) RT‐qPCR and western blot analysis of CSC‐like behavior related genes and proteins in MDA‐MB‐231 and MDA‐MB‐231 tumor spheres. Data are presented as mean ± SD from three independent experiments. Statistical significance was determined by student's t‐test (^*^p < 0.05, ^**^p < 0.01, ^***^p < 0.001). (B‐C) RT‐qPCR and western blotting analysis of USP30 overexpression and knockdown efficiency in MDA‐MB‐231 and BT549 cells infected with lentiviruses carrying empty vector, USP30, sh‐Con or USP30 shRNA. (D) MDA‐MB‐231 cells with USP30 overexpression or USP30 knockdown were exposed to different concentrations of Cisplatin for 72 h, and the cell viability was determined by CCK‐8. (E) MDA‐MB‐231 cells infected with lentiviruses carrying empty vector, USP30, USP30‐NLS2m or USP30‐C77S were exposed to different concentrations of Cisplatin for 72 h, and the cell viability was determined by CCK‐8. Data were normalized to the control (set as 100%) and are presented as means ± SD from three independent experiments. (F) ALDH activity of MDA‐MB‐231 spheres transfected with empty vector, USP30, USP30‐NLS2m or USP30‐C77S was assessed by flow cytometry. (G) Representative images of MDA‐MB‐231 sphere formation after transfection with different plasmids.
**Figure S4**: Subcellular localization of USP30 determines its function. (A‐B) Quantification of gray values for immunoblot bands to assess the effect of USP30 truncation mutations on USP30 subcellular localization. (C) Western blot analysis of USP30 expression in whole cell extracts, cytoplasm, mitochondria, and nucleus of MDA‐MB‐231 cells transfected with His‐tagged USP30 truncation mutants. (D) Immunofluorescence images showing the localization of USP30 in MDA‐MB‐231 cells transfected with USP30 truncation mutants. (E) Immunofluorescence images showing TOM20‐marked mitochondrial and LAMP1‐marked lysosomes. White dotted‐line boxes indicate regions magnified in insets. Scale bars: 10 µm. (F) Quantification of mean fluorescence intensity of TOM20. Data are presented as mean ± SD, statistical significance was determined by one‐way ANOVA (^*^p < 0.05, ^**^p < 0.01, ^***^p < 0.001). (G) Immunoblots of TIM23 and TOM20 in MDA‐MB‐231 cells transfected with USP30 truncation mutants, treated with or without CCCP (10 µM, 24 h).
**Figure S5**: Subcellular distribution of USP30 determines its role in inhibiting breast cancer stemness and chemoresistance. (A) MDA‐MB‐231 cells with USP30 or its subcellular localization mutants overexpressed were exposed to different concentrations of Adriamycin for 72 h, and the cell viability was determined by CCK‐8. Data were normalized to the vector control (set as 100%) and are presented as means ± SD from three independent experiments. (B) MDA‐MB‐231 cells with USP30 or its subcellular localization mutants overexpressed were exposed to different concentrations of cisplatin for 72 h, and the cell viability was determined by CCK‐8. (C‐D) Sphere formation ability of MDA‐MB‐231 cells with USP30 or its subcellular localization mutants overexpressed. (E) Western blot analysis of cancer stemness and chemoresistance markers in MDA‐MB‐231 cells with USP30 or its subcellular localization mutants overexpressed.
**Figure S6**: Nuclear transcription factor TCF/LEF1 is a *de novo* substrate of USP30. (A) Mass spectrometry analysis of subcellular localization of USP30‐interacting proteins. (B) Co‐IP assay showed the interaction between endogenous USP30 and LEF1 in MDA‐MB‐231 cells. (C) MDA‐MB‐231 cells transfected with His‐USP30 or His‐USP30‐C77S, Flag‐LEF1 and HA‐Ub were lysed and immunoprecipitated with anti‐Flag beads, followed by immunoblotting with indicated antibodies. (D) HEK293 cells transfected with His‐USAP30, Flag‐LEF1and HA‐Ub (K6, K11, K48, K63) were lysed, immunoprecipitated with anti‐Flag beads and blotted with indicated antibodies. (E) CHX chase analysis of LEF1 and USP30 proteins level in MDA‐MB‐231 cells with USP30 overexpression or knockdown. (F) RT‐qPCR analysis of LEF1 expression in MDA‐MB‐231 cells infected with lentiviruses carrying empty vector, USP30, sh‐Con, or USP30 shRNA. (G) HEK293 cells transfected with His‐USP30 or His‐vector. β‐catenin were lysed and immunoprecipitated using anti‐β‐catenin, followed by immunoblotting with anti‐P300, anti‐BCL9, and anti‐PYGO. (H) RT‐qPCR analysis of Wnt target gene expression in HEK293 cells transfected with His‐vector, His‐USP30, or USP30 NLS mutants. Data are presented as mean ± SD from three independent experiments, statistical significance was determined by one‐way ANOVA (^*^p < 0.05, ^**^p < 0.01, ^***^p < 0.001).
**Figure S7**: USP30‐mediated deubiquitination of LEF1 suppresses its transcriptional activity and cancer stemness in TNBC cells. (A) Six lysine residues (K374, K375, K376, K377, K379, K382) in LEF1 were mutated to arginine R. HEK293 cells were transfected with plasmids as indicated. The lysates were immunoprecipitated with anti‐Flag beads and immunoblotted with anti‐ubiquitin to assess the ubiquitination levels. (B) HEK293 cells were co‐transfected with His‐USP30 or His‐vector, HA‐Ub, and Flag‐LEF1, Flag‐LEF1‐K379A, or Flag‐LEF1‐K382A. The lysates were immunoprecipitated with anti‐Flaag beads and immunoblotted with anti‐ubiquitin to assess the ubiquitination levels. (C) Dual luciferase reporter gene assays to assess the transcriptional activity of the LEF1 gene in MDA‐MB‐231 cells co‐transfected with His‐vector or His‐USP30 and Flag‐LEF1, or LEF1 K379 K382 mutants. (D) MDA‐MB‐231 cells with LEF1 or LEF1‐K379A/K382A overexpression were exposed to different concentrations of Cisplatin for 72 h, and the cell viability was determined by CCK‐8. Data were normalized to the LEF1 control (set as 100%) and are presented as means ± SD from three independent experiments.
**Figure S8**: The clinical significance and tumor‐suppressive function of nuclear USP30 in breast cancer. (A) Comparison of USP30 expression between TNBC (n = 116) and other breast cancer subtypes (n = 862) obtained from the TCGA‐BRCA database. (B‐C) The expression of USP30 in TNBC and other types of breast cancer counted from the GEO datasets GSE41313 and GSE76275. p < 0.001, by Mann‐Whitney U test. (D) UMAP plot showing grouping based on USP30 expression levels. (E) GSEA revealed enrichment of differentially expressed genes in the EMT pathway. (F‐G) The expression of USP30 in estrogen receptor‐negative and estrogen receptor‐positive breast cancer counted from the GEO public microarray datasets GSE45268 and GSE21653. p < 0.001, by Mann‐Whitney U test.
**Figure S9**: Overexpression of nuclear USP30 reduces lung metastatic burden in TNBC models. (A) Representative bioluminescent images of lung metastases. (B) Representative bioluminescent images of lung metastases of MDA‐MB‐231‐Luc cells in mice. (C) Representative bioluminescent images of lung metastases of 4T1‐Luc cells in mice.
**Table S1**: Sequences of primer and siRNA.
**Table S2**: Antibodies used in this study.

## Data Availability

The publicly available data are provided in TCGA, GEO, METABRIC, and CBioportal (http://www.cbioportal.org/) database. All the data supporting the findings of this study are available within the article and its supporting information files or from the corresponding authors upon reasonable request.
