# Supplementary material for: mTORC1‐USP30‐LEF1 Cascade Regulates Cancer Stemness and Malignant Progression Through Mitonuclear Crosstalk
Source: MedComm (2020). 2025 Nov 24;6(12):e70499. doi: 10.1002/mco2.70499 (PMC12644247; doi:10.1002/mco2.70499)
Supplement: Supplementary file 1 — Figure S1: Phosphorylation at serine 104 regulates the nuclear translocation of USP30. (A) Western blot analysis of USP30 in cytoplasmic (Cyto) and nuclear (Nuc) fractions of HEK293, HepG2, and HeLa cells. Ratios of cytoplasmic‐to‐nuclear USP30 were quantified using image J. (B) Subcellular fractionation and immunoblotting were performed, and Image J was used to analyze the relative protein expression of USP30 in nuclei and mitochondria at different time points of serum deprivation. Data presented are derived from three independent experiments. (C) Immunofluorescence staining and quantification of mean fluorescence intensity of USP30 in MDA‐MB‐231 cells after glucose restriction (4 h) and serum deprivation (4 h). Scale bar, 15 µm. Data are presented as mean ± SD, statistical significance was determined by one‐way ANOVA (∗∗∗p < 0.001).(C) Representative USP30‐interacting proteins identified by mass spectrometry. (D‐E) Western blot analysis of USP30 subcellular localization in MDA‐MB‐231 cells treated with D4476 (CKI inhibitor: 20 µM, 12 h), Silmitasertib (CKII inhibitor: 1 µM, 12 h), H 89 2HCL (PKA inhibitor: 20 µM, 2 h), and SB202190(FHPI) (MAPK inhibitor: 50 mM, 24 h). (F) Co‐IP assay showing the interaction between endogenous USP30 and mTOR in MDA‐MB‐231 cells. (G) Co‐IP analysis of P‐S/T levels of USP30 in different cellular fractions. Figure S2: Gene co‐expression analysis of USP30 in breast cancer. (A) Gene co‐expression analysis between USP30 and CSC‐like behavior related genes in breast cancer cohorts from the CBioportal database. (B‐F) Gene co‐expression analysis between USP30 and MMP9, MYC, OCT4, ALDH1A3, CD133 in breast cancer cohorts. Figure S3: USP30 inhibits cancer stemness and chemoresistance in TNBC cells. (A) RT‐qPCR and western blot analysis of CSC‐like behavior related genes and proteins in MDA‐MB‐231 and MDA‐MB‐231 tumor spheres. Data are presented as mean ± SD from three independent experiments. Statistical significance was determined by student' [file MCO2-6-e70499-s001.docx]

**Supporting Information**

**mTORC1-USP30-LEF1 Cascade Regulates Cancer Stemness and Malignant Progression Through Mitonuclear Crosstalk**

Xiaolin Li^1#^, Haowei Zhang^1#^, Jia Li^1^, Cheng Luo^1^, Zijian Yang^2^, Jin Cai^1^, Li Xia^1^, Qian Peng^1^, Yapei Jiang^1^, Ruonan Wang^1^, Shiyue Yang^1^, Hongli Zeng^1^, Yuetong Li^1^, Haitao Yang^1^, Tong Gao^1^, Weidong Xie^1^, Yaou Zhang^1^, Naihan Xu^1,^ ^3*^

1. State Key Laboratory of Chemical Oncogenomics, Institute of Biopharmaceutical and Health Engineering, Tsinghua Shenzhen International Graduate School, Tsinghua University, Shenzhen 518055, China
2. Department of Breast and Thyroid Surgery, Peking University Shenzhen Hospital, Shenzhen 518034, China
3. School of Food and Drug, Shenzhen Polytechnic University, Shenzhen 518055, China

^#^ Xiaolin Li and Haowei Zhang contributed equally to this work.

* Corresponding author

**^*^**Correspondence:

Professor Naihan Xu

School of Food and Drug, Shenzhen Polytechnic University, Shenzhen 518055, China

Email: xu_naihan@szpu.edu.cn

Running title: mTORC1-USP30-LEF1 regulates malignant progression

**Supplementary Materials and Methods**

1. **Mass spectrometry analysis of USP30 protein interactions**

The mass spectrometry analysis was performed by PTM Bio (Hangzhou, China). Briefly, USP30 and its interacting proteins were immunoprecipitated from MDA-MB-231 cell lysates using USP30 antibodies. The enriched protein complexes were separated by SDS-PAGE, and the target band (~55 kDa) was excised for in-gel tryptic digestion. The resulting peptides were analyzed by nanoLC-MS/MS using a Q Exactive HF-X mass spectrometer (Thermo Fisher Scientific) coupled to an EASY-nLC 1200 system. Data were processed with MaxQuant against the UniProt human database. High-confidence interactors were filtered at 1% FDR. For bioinformatics analysis, the identified potential USP30-interacting proteins were subjected to Gene Ontology (GO) enrichment analysis, KEGG pathway enrichment analysis, and subcellular localization prediction analysis.

1. **Single cell RNA-seq data analysis**

Single-cell RNA-sequencing data (ArrayExpress accession: E-MTAB-8107) were processed using Seurat v4.3.0. Initial quality control involved filtering cells with fewer than 400 detected genes or over 20% mitochondrial gene content. Potential doublets were identified and removed using DoubletFinder v2.0.3. Subsequent normalization, identification of highly variable genes, and clustering were carried out using the default parameters and standard workflow provided by Seurat. To integrate data across different samples, we used Harmony v1.2.3 for batch effect correction.

To identify marker genes for each cluster, we performed differential gene expression analysis using Seurat’s FindAllMarkers function. Genes with an adjusted p-value < 0.05, expression in more than 25% of cells, and |log₂(fold change)| > 0.25 were considered as candidate marker genes. Cell type annotation was then performed based on the expression patterns of these marker genes, using references from published literature and manual curation to assign biological identities to each cluster.

**Supplementary Figures**


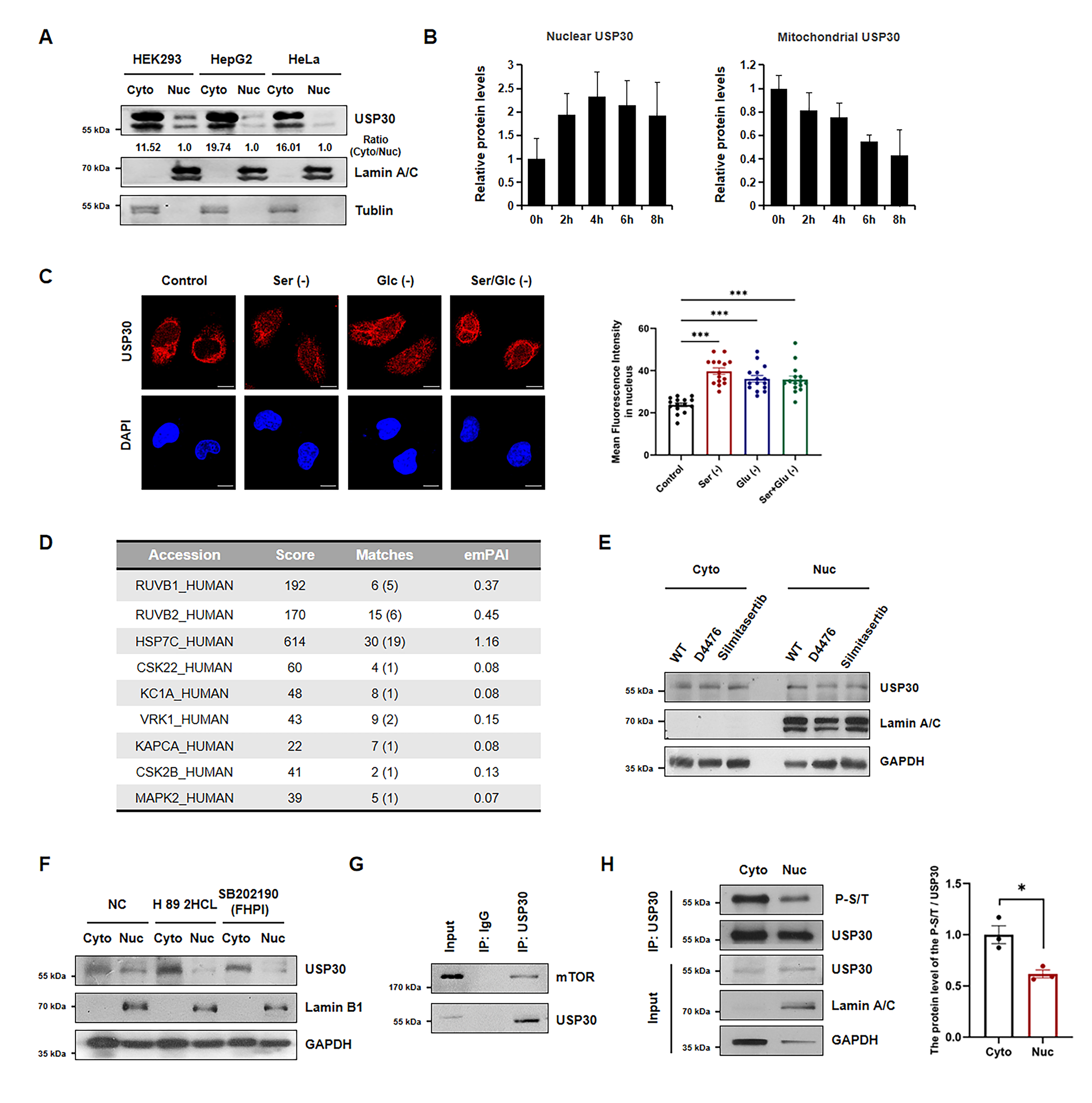


**Figure S1.** **Phosphorylation at serine 104** **regulates the nuclear translocation of USP30.** (A) Western blot analysis of USP30 in cytoplasmic (Cyto) and nuclear (Nuc) fractions of HEK293, HepG2, and HeLa cells. Ratios of cytoplasmic-to-nuclear USP30 were quantified using image J. (B) Subcellular fractionation and immunoblotting were performed, and Image J was used to analyze the relative protein expression of USP30 in nuclei and mitochondria at different time points of serum deprivation. Data presented are derived from three independent experiments. (C) Immunofluorescence staining and quantification of mean fluorescence intensity of USP30 in MDA-MB-231 cells after glucose restriction (4 h) and serum deprivation (4 h). Scale bar, 15 μm. Data are presented as mean ± SD, statistical significance was determined by one-way ANOVA (^∗∗∗^p < 0.001).(C) Representative USP30-interacting proteins identified by mass spectrometry. (D-E) Western blot analysis of USP30 subcellular localization in MDA-MB-231 cells treated with D4476 (CKⅠ inhibitor: 20 μM, 12 h), Silmitasertib (CKⅡ inhibitor: 1 μM, 12 h), H 89 2HCL (PKA inhibitor: 20 μM, 2 h), and SB202190(FHPI) (MAPK inhibitor: 50 mM, 24 h). (F) Co-IP assay showing the interaction between endogenous USP30 and mTOR in MDA-MB-231 cells. (G) Co-IP analysis of P-S/T levels of USP30 in different cellular fractions.

**
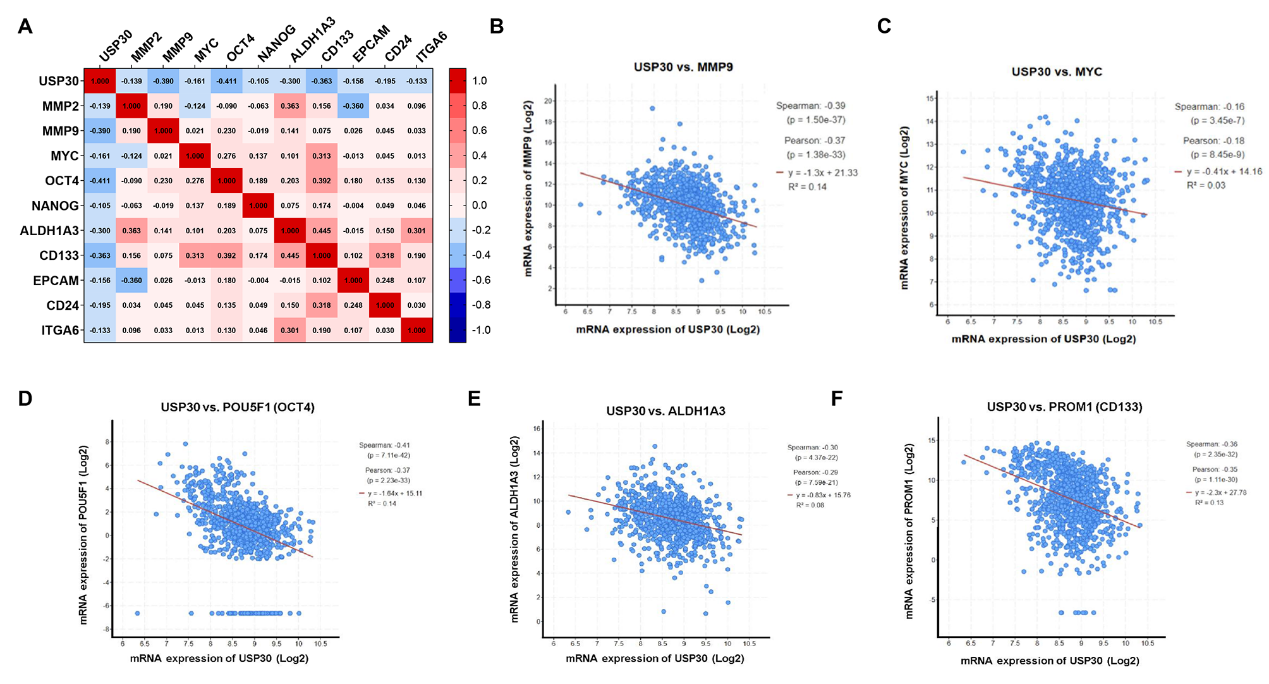
**

**Figure S2.** **Gene co-expression analysis of USP30 in breast cancer.** (A) Gene co-expression analysis between USP30 and CSC-like behavior related genes in breast cancer cohorts from the CBioportal database. (B-F) Gene co-expression analysis between USP30 and MMP9, MYC, OCT4, ALDH1A3, CD133 in breast cancer cohorts.

**
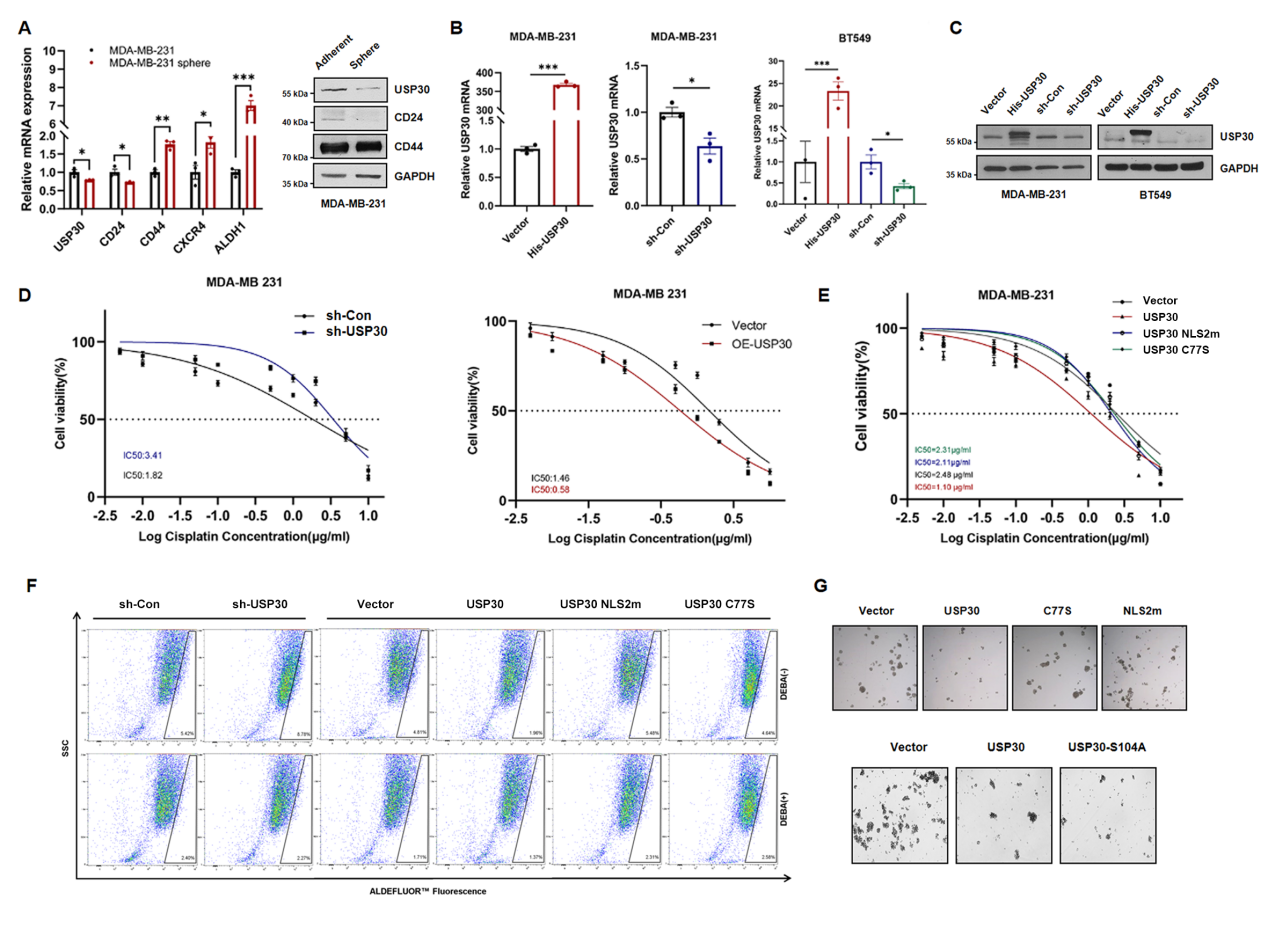
**

**Figure S3. USP30 inhibits cancer stemness and chemoresistance in TNBC cells.** (A) RT-qPCR and western blot analysis of CSC-like behavior related genes and proteins in MDA-MB-231 and MDA-MB-231 tumor spheres. Data are presented as mean ± SD from three independent experiments. Statistical significance was determined by student’s t-test (^*^p < 0.05, ^**^p < 0.01, ^***^p < 0.001). (B-C) RT-qPCR and western blotting analysis of USP30 overexpression and knockdown efficiency in MDA-MB-231 and BT549 cells infected with lentiviruses carrying empty vector, USP30, sh-Con or USP30 shRNA. (D) MDA-MB-231 cells with USP30 overexpression or USP30 knockdown were exposed to different concentrations of Cisplatin for 72 h, and the cell viability was determined by CCK-8. (E) MDA-MB-231 cells infected with lentiviruses carrying empty vector, USP30, USP30-NLSm or USP30-C77S were exposed to different concentrations of Cisplatin for 72 h, and the cell viability was determined by CCK-8. Data were normalized to the control (set as 100%) and are presented as means ± SD from three independent experiments. (F) ALDH activity of MDA-MB-231 spheres transfected with empty vector, USP30, USP30-NLSm or USP30-C77S was assessed by flow cytometry. (G) Representative images of MDA-MB-231 sphere formation after transfection with different plasmids.

**
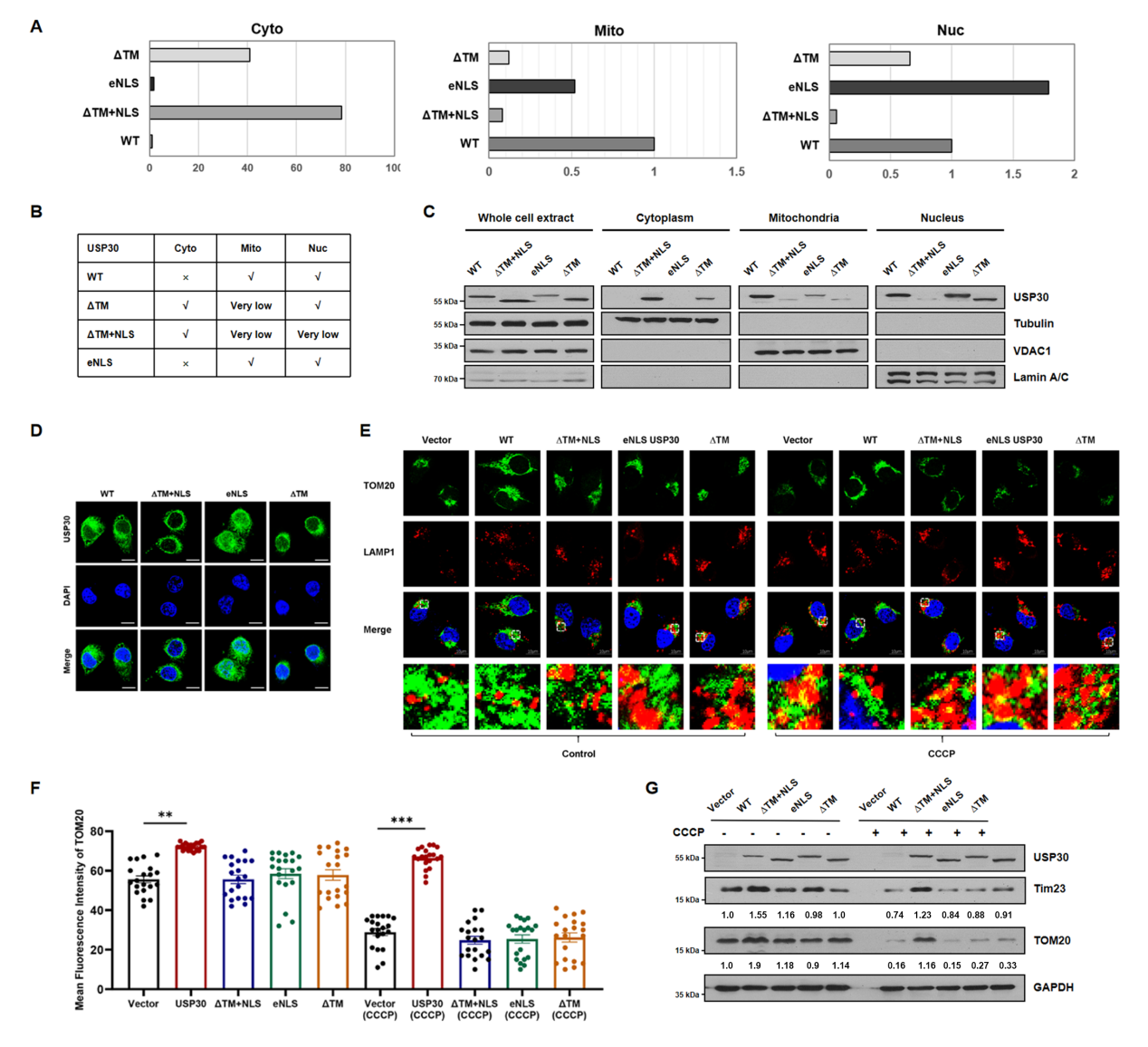
**

**Figure S4. Subcellular localization of USP30 determines its function.** (A-B) Quantification of gray values for immunoblot bands to assess the effect of USP30 truncation mutations on USP30 subcellular localization. (C) Western blot analysis of USP30 expression in whole cell extracts, cytoplasm, mitochondria, and nucleus of MDA-MB-231 cells transfected with His-tagged USP30 truncation mutants. (D) Immunofluorescence images showing the localization of USP30 in MDA-MB-231 cells transfected with USP30 truncation mutants. (E) Immunofluorescence images showing TOM20-marked mitochondrial and LAMP1-marked lysosomes. White dotted-line boxes indicate regions magnified in insets. Scale bars: 10 μm. (F) Quantification of mean fluorescence intensity of TOM20. Data are presented as mean ± SD, statistical significance was determined by one-way ANOVA (^*^p < 0.05, ^**^p < 0.01, ^***^p < 0.001). (G) Immunoblots of TIM23 and TOM20 in MDA-MB-231 cells transfected with USP30 truncation mutants, treated with or without CCCP (10 μM, 24 h).


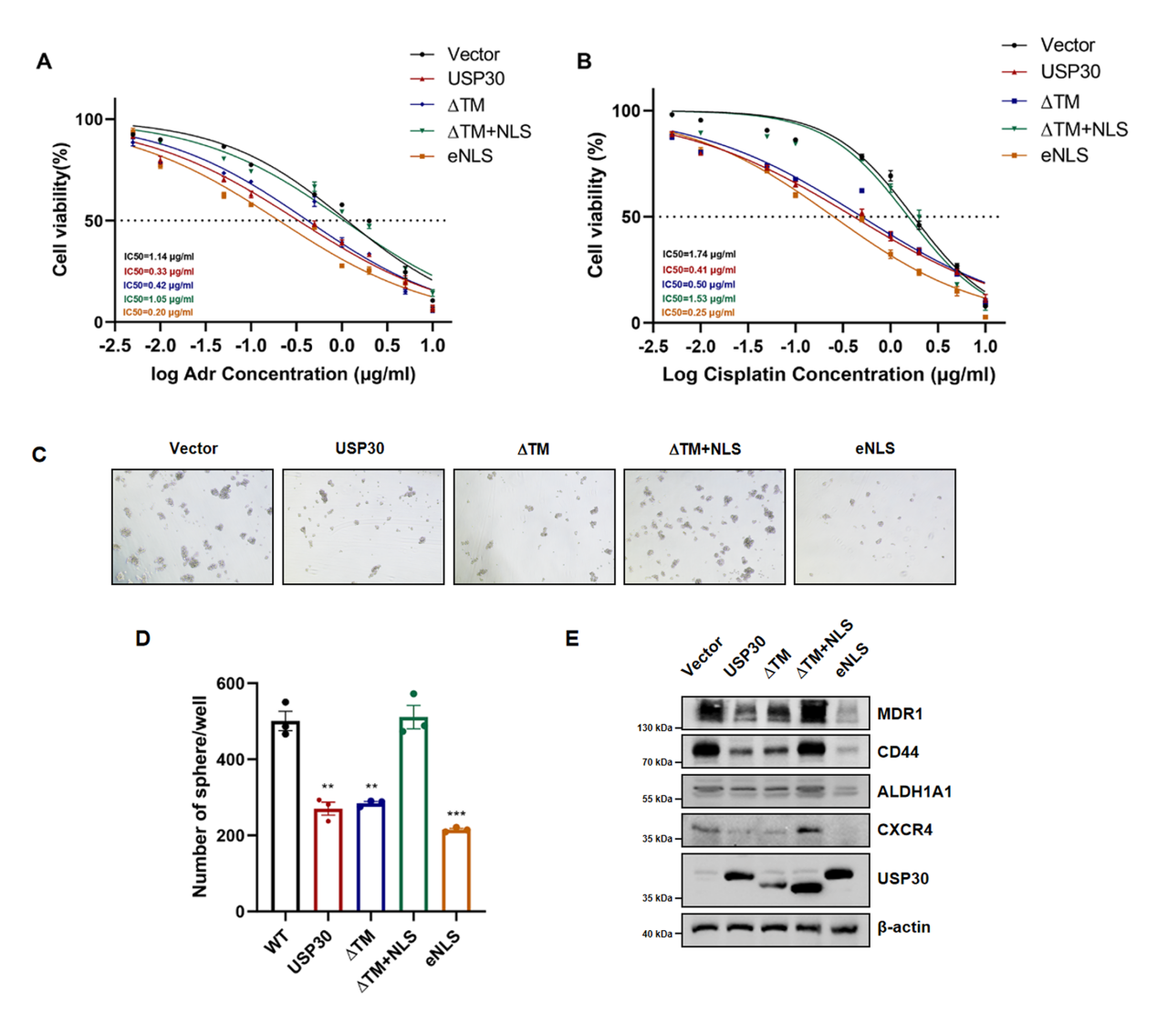


**Figure S5.** **Subcellular distribution of USP30 determines its role in inhibiting breast cancer stemness and chemoresistance.** (A) MDA-MB-231 cells with USP30 or its subcellular localization mutants overexpressed were exposed to different concentrations of Adriamycin for 72 h, and the cell viability was determined by CCK-8. Data were normalized to the vector control (set as 100%) and are presented as means ± SD from three independent experiments. (B) MDA-MB-231 cells with USP30 or its subcellular localization mutants overexpressed were exposed to different concentrations of cisplatin for 72 h, and the cell viability was determined by CCK-8. (C-D) Sphere formation ability of MDA-MB-231 cells with USP30 or its subcellular localization mutants overexpressed. (E) Western blot analysis of cancer stemness and chemoresistance markers in MDA-MB-231 cells with USP30 or its subcellular localization mutants overexpressed.

**
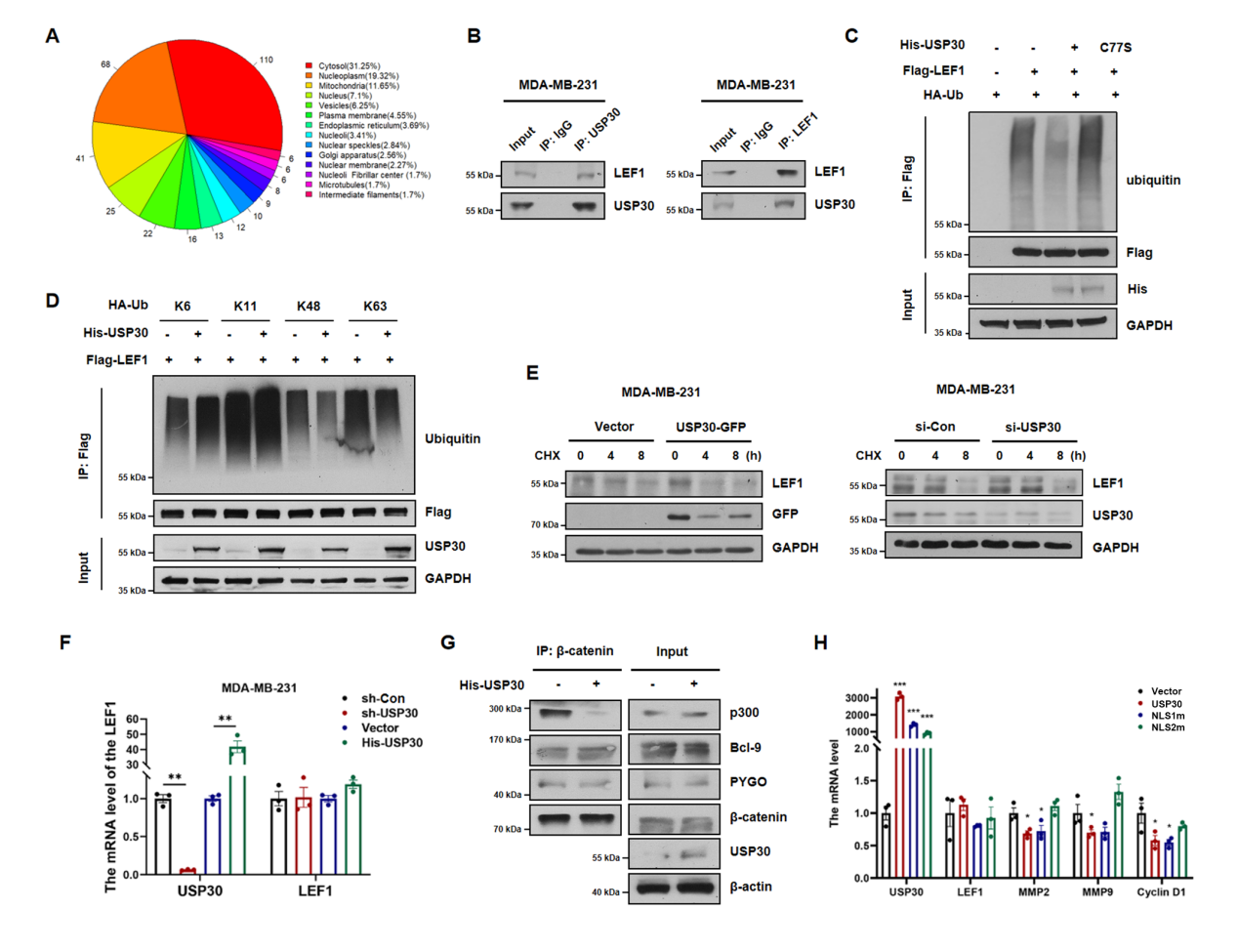
**

**Figure S6. Nuclear transcription factor TCF/LEF1 is a *de novo* substrate of USP30.** (A) Mass spectrometry analysis of subcellular localization of USP30-interacting proteins. (B) Co-IP assay showed the interaction between endogenous USP30 and LEF1 in MDA-MB-231 cells. (C) MDA-MB-231 cells transfected with His-USP30 or His-USP30-C77S, Flag-LEF1 and HA-Ub were lysed and immunoprecipitated with anti-Flag beads, followed by immunoblotting with indicated antibodies. (D) HEK293 cells transfected with His-USAP30, Flag-LEF1and HA-Ub (K6, K11, K48, K63) were lysed, immunoprecipitated with anti-Flag beads and blotted with indicated antibodies. (E) CHX chase analysis of LEF1 and USP30 proteins level in MDA-MB-231 cells with USP30 overexpression or knockdown. (F) RT-qPCR analysis of LEF1 expression in MDA-MB-231 cells infected with lentiviruses carrying empty vector, USP30, sh-Con, or USP30 shRNA. (G) HEK293 cells transfected with His-USP30 or His-vector. β-catenin were lysed and immunoprecipitated using anti-β-catenin, followed by immunoblotting with anti-P300, anti-BCL9, and anti-PYGO. (H) RT-qPCR analysis of Wnt target gene expression in HEK293 cells transfected with His-vector, His-USP30, or USP30 NLS mutants. Data are presented as mean ± SD from three independent experiments, statistical significance was determined by one-way ANOVA (^*^p < 0.05, ^**^p < 0.01, ^***^p < 0.001).

**
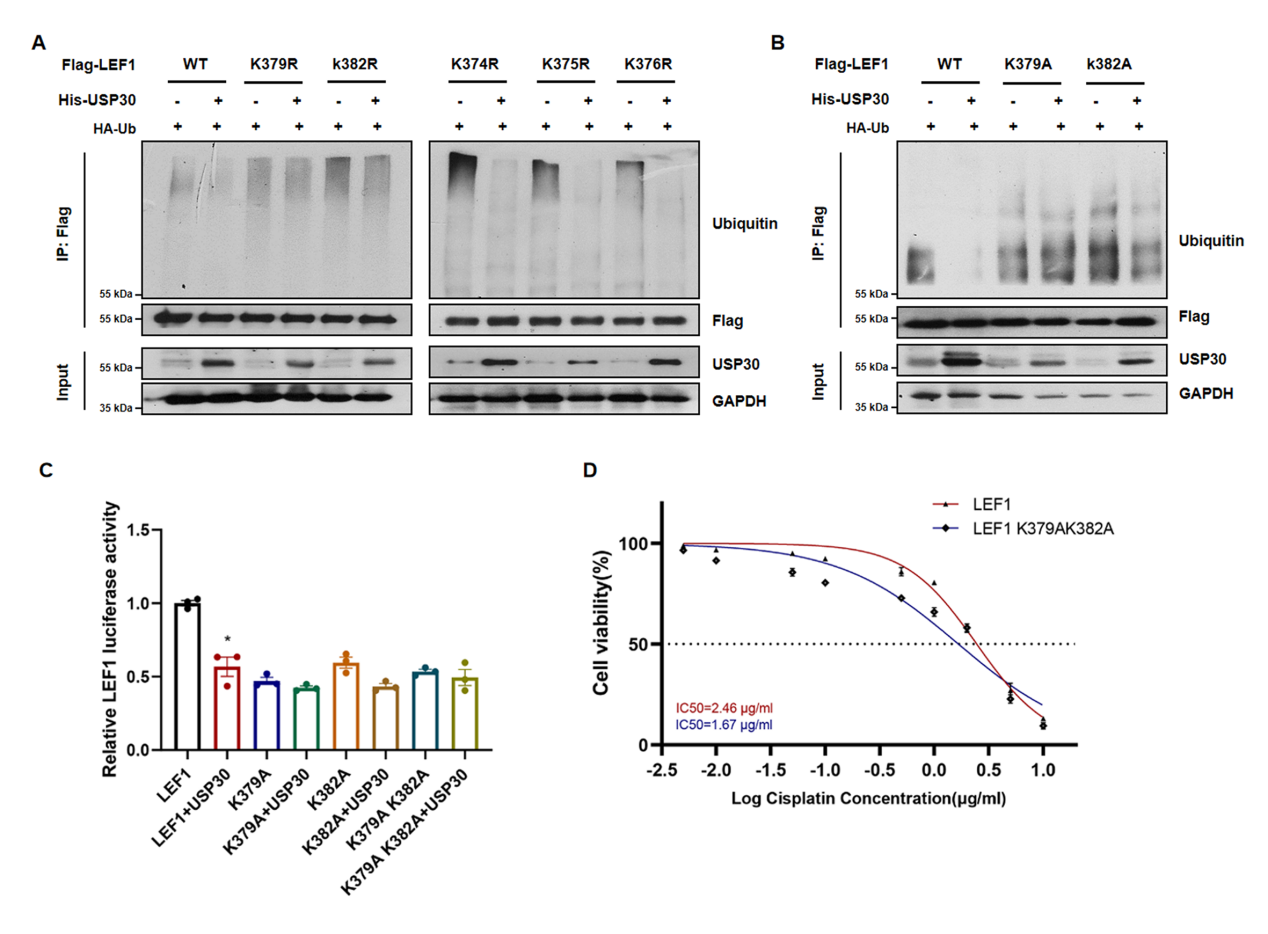
**

**Figure S7.** **USP30-mediated deubiquitination of LEF1 suppresses its transcriptional activity and cancer stemness in TNBC cells.** (A) Six lysine residues (K374, K375, K376, K377, K379, K382) in LEF1 were mutated to arginine R. HEK293 cells were transfected with plasmids as indicated. The lysates were immunoprecipitated with anti-Flag beads and immunoblotted with anti-ubiquitin to assess the ubiquitination levels. (B) HEK293 cells were co-transfected with His-USP30 or His-vector, HA-Ub, and Flag-LEF1, Flag-LEF1-K379A, or Flag-LEF1-K382A. The lysates were immunoprecipitated with anti-Flaag beads and immunoblotted with anti-ubiquitin to assess the ubiquitination levels. (C) Dual luciferase reporter gene assays to assess the transcriptional activity of the LEF1 gene in MDA-MB-231 cells co-transfected with His-vector or His-USP30 and Flag-LEF1, or LEF1 K379 K382 mutants. (D) MDA-MB-231 cells with LEF1 or LEF1-K379A/K382A overexpression were exposed to different concentrations of Cisplatin for 72 h, and the cell viability was determined by CCK-8. Data were normalized to the LEF1 control (set as 100%) and are presented as means ± SD from three independent experiments.


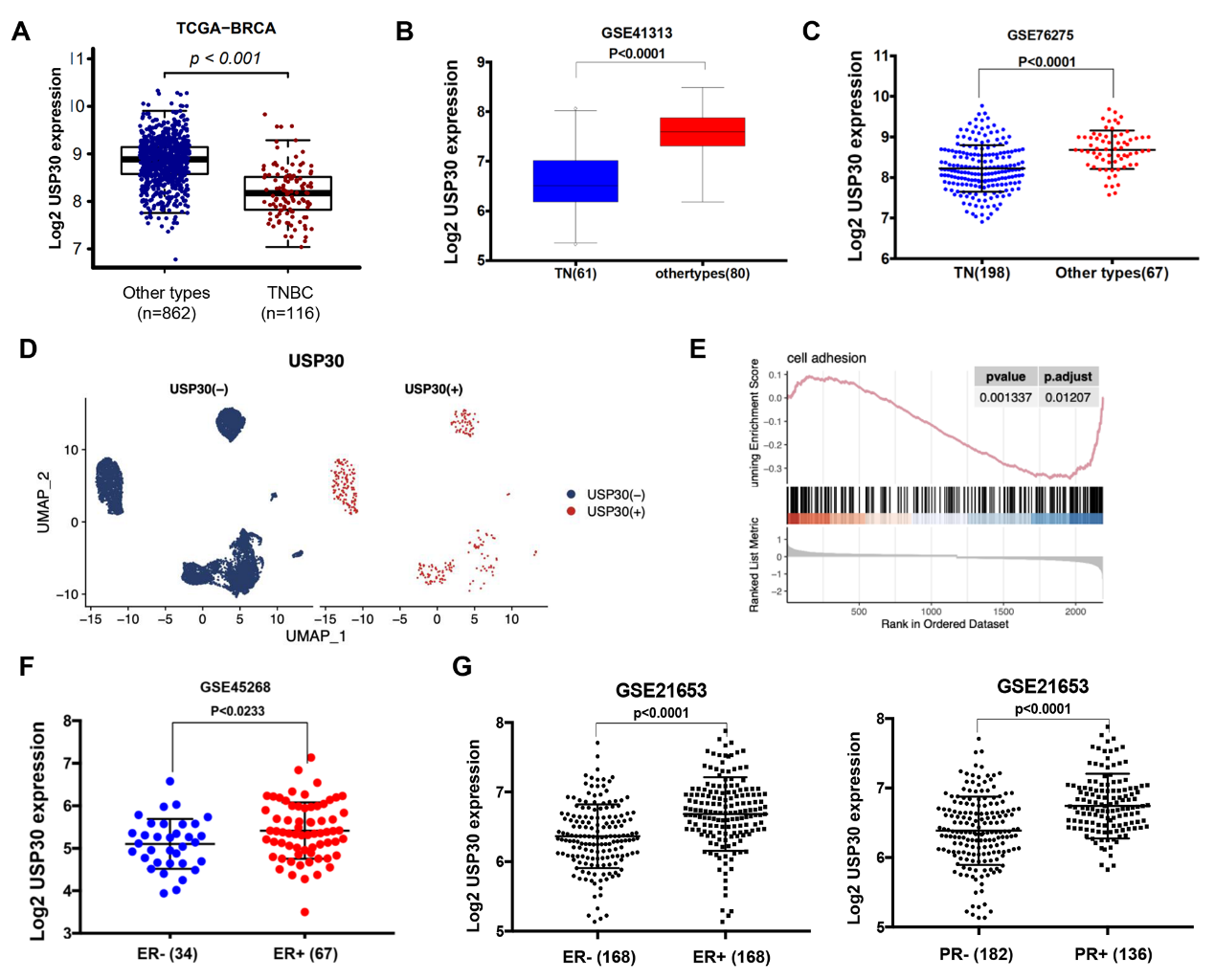


**Figure S8. The clinical significance and tumor-suppressive function of nuclear USP30 in breast cancer.** (A) Comparison of USP30 expression between TNBC (n=116) and other breast cancer subtypes (n=862) obtained from the TCGA-BRCA database. (B-C) The expression of USP30 in TNBC and other types of breast cancer counted from the GEO datasets GSE41313 and GSE76275. p < 0.001, by Mann-Whitney U test. (D) UMAP plot showing grouping based on USP30 expression levels. (E) GSEA revealed enrichment of differentially expressed genes in the EMT pathway. (F-G) The expression of USP30 in estrogen receptor-negative and estrogen receptor-positive breast cancer counted from the GEO public microarray datasets GSE45268 and GSE21653. p < 0.001, by Mann-Whitney U test.


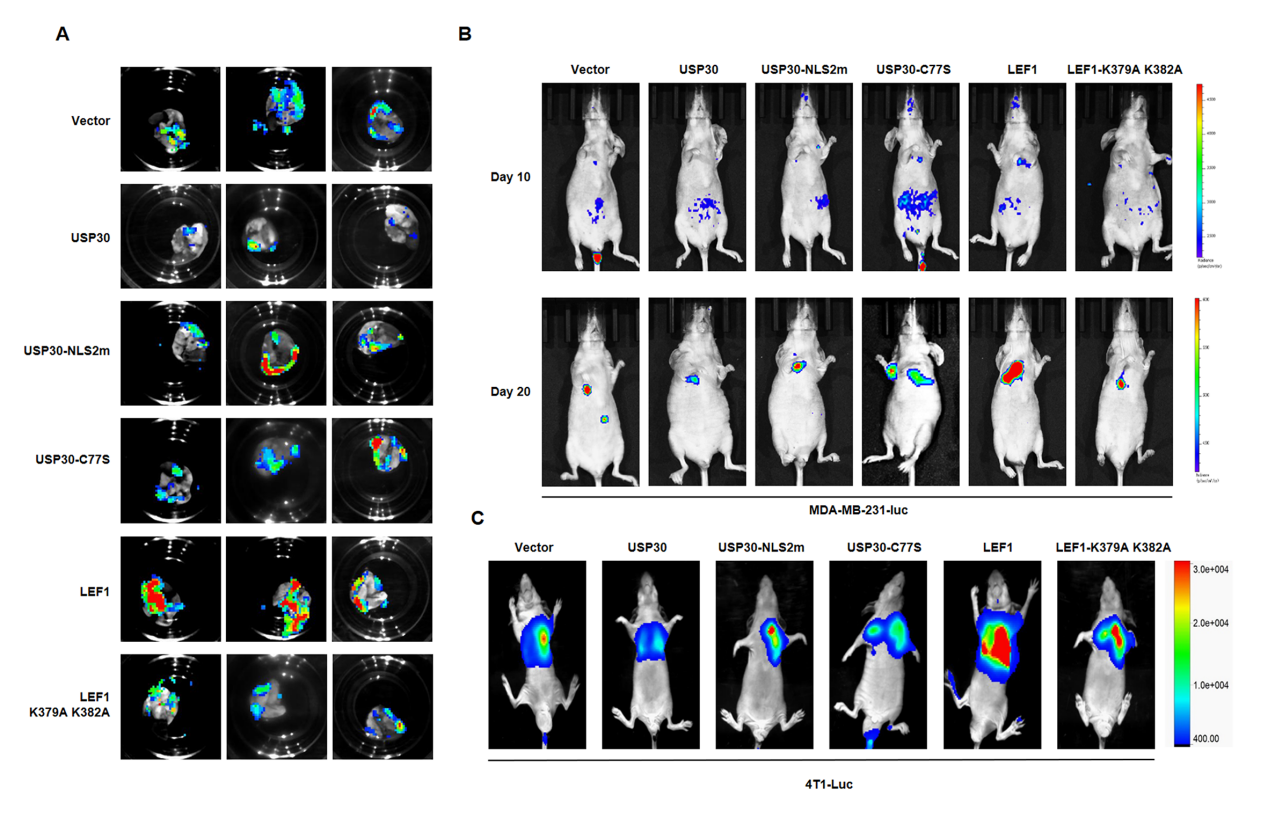


**Figure S9. Overexpression of nuclear USP30 reduces lung metastatic burden in TNBC models.** (A) Representative bioluminescent images of lung metastases. (B) Representative bioluminescent images of lung metastases of MDA-MB-231-Luc cells in mice. (C) Representative bioluminescent images of lung metastases of 4T1-Luc cells in mice.

**Supplementary Tables**

| **Gene** | **Sequences** |
| --- | --- |
| USP30 | F, 5’-GTCACCTCACCCTACATCCAA-3’  R, 5’-TGTGGCGGCTGGAATACTTAG-3’ |
| LEF1 | F, 5’-AGAACACCCCGATGACGGA-3’  R, 5’-GGCATCATTATGTACCCGGAAT-3’ |
| MMP2 | F, 5’-GAGGTCGCTTTCTTTGCCATCT-3’  R, 5’-AGCGACTCCATCTTGAACAGG-3’ |
| MMP9 | F, 5’-CGGCATCGGGCAGGGTCT-3’  R, 5’-CACTGTATCCTTGACCTTCTTTCTGG-3’ |
| CCND1 | F,5’-GGATGGAGTTGTCGGTGTAGATG-3’  R,5’-AGGAACAGAAGTGCGAGGAGG-3’ |
| C-myc | F, 5’-CTTCTCTCCGTCCTCGGATTCT-3’  R-5’-GAAGGTGATCCAGACTCTGACCTT-3’ |
| CD44 | F, 5’-CTGCCGCTTTGCAGGTGTA-3’  R-5’-CATTGTGGGCAAGGTGCTATT-3’ |
| CD24 | F, 5’-GAGAGATAACCCTGCCCGAG-3’  R-5’-CAAAAGAAAAGTCCGCGCCT-3’ |
| CXCR4 | F, 5’-ACTACACCGAGGAAATGGGCT-3’  R-5’-GCCGCTCTGGTAGTGCTG-3’ |
| SOX2 | F, 5’-TGGGTTCGGTGGTCAAGTC-3’  R-5’-TCCACAGAAACAACATCG-3’ |
| ALDH1 | F, 5’-TGCAGGTTGGGCTGACAA-3’  R-5’-TCCACAGAAACAACATCG-3’ |
| si-USP30#1 | Sense (5’-3’) GCUGCUUGUUGGAUGUCUU  Antiaense (5’-3’) AAGACAUCCAACAAGCAGG |
| si-USP30#2 | Sense (5’-3’) CAAAUUACCUGCCGCACAA  Antiaense (5’-3’) UUGUGCGGCAGGUAAUUUG |
| si-NC | Sense (5’-3’) UUCUCCGAACGUGUCACGUTT  Antiaense (5’-3) ACGUGACACGUUAGAATT |

Table S1. Sequences of primer and siRNA.

| **Antibodies** | |  |
| --- | --- | --- |
| anti-USP30 (abcam, #ab314749) | anti-LEF1 (CST, #2230) |  |
| anti-GAPDH (proteintech, #10494-1-AP) | anti-TOM20 (CST, #42406) |  |
| anti-LaminA/C (CST, #4777) | anti-LAMP1 (CST, #9091) |  |
| anti-Lamin B1 (CST, #13435) | anti-Tim23 (proteintech, #11123-1-AP) |  |
| anti-Tubulin (CST, #2125) | anti-HA (abcam, #ab1424) |  |
| anti-VDAC1 (CST, #4866) | anti-Flag (CST, #14793) |  |
| anti-β-actin (proteintech, #60008-1-lg) | anti-GFP (proteintech, #66002-1-lg) |  |
| anti-importin β1 (CST, #51186) | anti-His (proteintech, #66005-1-lg) |  |
| anti-importin α /KPNA2 (CST, #14372) | anti-Ubiquitin (CST, #3936) |  |
| anti-β-catenin (CST, #8480) | anti-CBP (CST, #7389) |  |
| anti-Acetyl-β-Catenin (Lys49) (CST, #9030) | anti-P300 (CST, #86377) |  |
| anti-MMP9 (CST, #3852) | anti-Acetylated-lysine (CST, #9441) |  |
| anti-MMP2 (CST, #40994) | anti-Bcl9 (CST, #15096) |  |
| anti-CyclinD1(CST, #2978) | anti-PYGO (abcam, #ab109001) |  |
| anti-USP30 (Senta, #H1021) | anti-IgG (CST #5873) |  |
| anti-MDR1(CST, #13342) | | anti-P-gp (proteintech, #22336-1-AP) |
| anti-CXCR4 (proteintech, #60042-1-Ig) | | anti-ALDH1A1 (proteintech, #15910-1-AP) |
| anti-CD24 (proteintech, #67627-1-Ig) | anti-CD44 (proteintech, #15675-1-AP) |  |

Table S2. Antibodies used in this study.
